# Characterization of anticariogenic mycosymbiotic fungi associated with the medicinal plant *Piper crocatum*

**DOI:** 10.1038/s41598-026-41703-z

**Published:** 2026-03-18

**Authors:** Syifa Zahara Kultsum Azmi, Dikdik Kurnia, Rianti Nurpalah, Dewi Peti Virgianti, Rizal Padilah, Luluatul Fuadatin Nafisah Ruswadi, Toto Subroto

**Affiliations:** 1 Department of Chemistry, Universitas Padjadjaran, Dipati Ukur, Bandung, 40132 West Java Indonesia; 2 Department of Health Sciences, Univesitas Bakti Tunas Husada, Letjen Mashudi, Tasikmalaya, 46196 West Java Indonesia

**Keywords:** Mycosimbiont1, Anticariogenic2, Numerical taxonomy3, ITS sequencing4, Biotechnology, Microbiology, Plant sciences

## Abstract

The escalating antibiotic resistance in oral pathogens, particularly *Streptococcus mutans*, necessitates the discovery of alternative bioactive scaffolds. While *Piper crocatum* (red betel) is an ethnobotanically significant plant, its symbiotic mycobiota remains a negligible niche compared to heavily mined species like *P. nigrum* or *P. betle*. This study explored the diversity of fungal syimbiont from *P. crocatum* as potential anticariogenic agents. Fungal samples were isolated from leaves collected across 13 locations in Tasikmalaya, Indonesia. Purification for each fungi was conducted using the hyphal tip transfer technique, yielding 66 axenic isolates. Phenetic characterization was employed as a dereplication strategy to select ten representative based on 33 morphotypes for screening. Similarity analysis was conducted using the Jaccard coefficient via the UPGMA, then visualized in RStudio using the ape and ggtree packages to generate a phenetic dendrogram. One representative isolate from each major cluster was selected for bioactivity screening, with priority given to isolates producing visible extracellular exudates. Results indicated a positive correlation between colony pigmentation and bioactivity, with isolate t5-059 exhibiting the strongest inhibition against *S. mutans* (21.5 mm). Molecular identification via ITS rDNA resolved the bioactive strains as *Colletotrichum truncatum* (t5-059), *Colletotrichum cliviae* (t-9052), *Torula canangae* (t10-062), and *Aspergillus clavatonanicus* (t1-007). The recurrence of these specific taxa across geographically heterogeneous sites supports the hypothesis of host filtering, where *P. crocatum* selects for a core microbiome. These findings highlight *P. crocatum* as a reservoir of unique fungal associates capable of producing potent metabolites for oral health applications.

**Supplementary Information:**

The online version contains supplementary material available at 10.1038/s41598-026-41703-z.

## Introduction

Dental caries (ICD-10: K02) is a chronic oral disease characterized by the demineralization of the dental matrix, mediated by acid-producing microbes within a biofilm. This process is driven by the consumption of fermentable carbohydrates, which are metabolized into acidic intermediates that cumulatively cause carious lesions^1^. In Indonesia, the 2023 National Health Survey (SKI) reported that approximately 8 out of 10 individuals suffer from dental caries^2^, with a mean DMF-T (Decayed, Missing, and Filled Teeth) index of 4.0 in the 24–35 age group. This index increases with age, highlighting a significant risk of edentulism in the elderly population.

Current management of dental caries depends on the stage of invasion and often involves systemic or topical antimicrobials, as well as surgical intervention. However, the empirical use of broad-spectrum antibiotics—such as amoxicillin, metronidazole, or clindamycin—for advanced stages (pulpitis) has raised concerns. Studies show that only about 12% of dentists prescribe antibiotics accurately, and the resulting misuse significantly contributes to antimicrobial resistance (AMR) and opportunistic infections^3^. The clinical necessity for novel antimicrobial agents is further underscored by the alarming escalation of antibiotic resistance among primary oral pathogens. Recent data indicates that *S. mutans* isolates exhibit critical resistance levels to conventional dental antibiotics, with resistance rates reaching 80% for amoxicillin, 86.6% for ampicillin, and a staggering 93.3% for tetracycline^4^. Such high prevalence of resistance—particularly against first-line β-lactam antibiotics—significantly compromises the efficacy of standard restorative and proactive treatments. This crisis necessitates an urgent shift toward bioprospecting for alternative bioactive scaffolds from natural reservoirs, such as the mycosymbionts of medicinal plants, which may offer untapped chemical diversity to circumvent current resistance mechanisms. Furthermore, the dynamic nature of Streptococcus adhesion and detachment mechanisms within the oral ecosystem^5^, necessitates the discovery of new agents capable of modulating these microbial interactions without triggering resistance^6^.

Ethnobotanical traditions provide a strategic starting point for this discovery. Red betel (*Piper crocatum* Ruiz & Pav.) has been culturally significant since the Neolithic era, particularly through the “menginang” (betel chewing) tradition in Indonesia. While practitioners believe it strengthens teeth and treats oral wounds, prolonged chewing is also associated with dental abrasion, periodontitis, and even oral precancerous lesions^7^. Therefore, rather than raw consumption, there is a clinical need to identify and develop standardized bioactive “lead compounds” from *P. crocatum* that maximize its medicinal benefits while eliminating the risks associated with traditional chewing practices. Previous research has identified phytochemicals such as crocatins, piperiamines, and stigmasterol in *P. crocatum*^8–10^. Crocatin A, which is active against *S. sanguinis*^11^, as well as Piperyamine A, Piperyamide A, and Stigmasterol, which have antifungal activity against *C. albicans* with very low MIC values (0.31%–0.62%)^9^. Furthermore, Piperidine and Stigmasterol compounds from red betel leaves have been shown in vitro to inhibit various oral pathogenic bacteria, including *S. mutans, S. sobrinus*, and *A. viscosus*^12–14^. Given that this host plant has such a complex chemical defense mechanism, exploring the mycosymbionts that inhabit its internal tissues is a strategic step. These mycosymbionts have the potential to have similar biosynthetic pathways or even produce more potent and stable derivative compounds than the metabolism of their host plants, thus offering a more sustainable biotechnological solution to address the future antimicrobial resistance crisis. However, the direct exploitation of medicinal plants for large-scale production faces significant hurdles, including long growth cycles, susceptibility to environmental fluctuations, and the ecological impact of intensive harvesting^15^. Consequently, finding sustainable and scalable alternatives for producing plant-derived bioactives is a critical challenge in modern drug discovery.

A promising but underexplored avenue lies in the mycosymbionts residing within the plant’s internal tissues. While the ecological roles of these mycosymbionts can be complex—ranging from mutualism to latent pathogenicity—their presence within the host’s internal niche often triggers the synthesis of secondary metabolites that are structurally or functionally analogous to plant compounds^16^. Unlike plant extracts, which are susceptible to seasonal and geographical fluctuations, these mycosymbionts can be cultivated in controlled environments, providing a sustainable and scalable source of bioactives. However, the mycosymbiotic diversity of many high-value medicinal plants remains a significant “black box” in the literature.

Despite reports of various fungi associated with the Piper genus, the mycosymbiotic diversity within the mesophyll tissues of *P. crocatum* and its specific potential against cariogenic pathogens remain largely unknown. This study addresses this gap by characterizing the diversity of mycosymbiotic fungi isolated from *P. crocatum* and screening their crude secondary metabolites for inhibitory activity against *S. mutans*. This research establishes an initial framework for identifying microbial-derived leads that can serve as ecological modulators in the management of dental caries. We employed a phenotype-based dereplication strategy followed by molecular identification to explore the diversity of these fungal partners. Furthermore, we evaluated the inhibitory potential of their crude secondary metabolites against *S. mutans* and utilized TLC profiling to characterize the chemical heterogeneity of the most bioactive isolates. This work establishes a foundation for identifying sustainable microbial.

## Methods

### Collection of plant material and selection criteria

Red betel (*Piper crocatum* Ruiz & Pav.) leaves were collected in Tasikmalaya, West Java, Indonesia. Samples were collected from public land where no specific permission was required. The species is not protected or endangered. Pathogen-free leaves of red betel were selected as the primary source of observation in this study. The leaves were screened visually to ensure the absence of necrosis, chlorosis, or any other visible signs of pathogenic infection^17^. Sampling was conducted from twelve distinct locations across the Tasikmalaya region, West Java, Indonesia, to capture a broad representation of the plant’s myosymbiotic fungal diversity. The distribution map of the sampling sites was generated using ArcGIS 10.8 (Esri, Redlands, CA, USA; https://www.esri.com/). Administrative boundary layers (province, district, and subdistrict levels) were obtained from the official Rupa Bumi Indonesia (RBI) dataset provided freely by the Geospatial Information Agency of Indonesia (Badan Informasi Geospasial, BIG; https://tanahair.indonesia.go.id/). The geographic coordinates of the sampling plants were recorded in the field using a handheld Global Positioning System (GPS) device and compiled into a comma-separated values (CSV) file containing latitude, longitude, and sample ID. The map was constructed using the Universal Transverse Mercator (UTM) projection, WGS 1984 datum, Zone 48S. Sampling points were visualized using distinct symbols and colors to differentiate each sample code (SM1001–SM1013). The administrative boundaries were displayed as polygon layers with contrasting outlines for clarity. Map elements including a north arrow, scale bar, and legend were added to improve readability.

## Surface sterilization and tissue preparation

All collected samples were placed in sterile petri dishes, transported under cooled conditions, and processed within 24 hours to minimize changes in the endogenous microbial community. Leaves were rinsed under running tap water to remove debris, followed by immersion in 70% (v/v) ethanol for 1 min, 1.3 mol/L sodium hypochlorite (NaOCl) for 3 min, and rinsed three times with sterile distilled water^18^. The abaxial surface of each leaf was oriented upward (inverted position), and a longitudinal medial section was excised along the midrib (primary vein) using a sterile scalpel blade (no. 11). Sections were prepared with 2.5 mm spacing on either side of the midrib, resulting in a 5 mm-wide central strip. This strip was then transversely subdivided into three segments, each measuring 2.5 mm in length, to obtain explants from mesophyll tissue.

## Isolation of mycosymbiotic fungi

Mycosymbiotic fungal isolation was carried out using a direct planting method. Three leaf explants were aseptically placed on the periphery of solidified Sabouraud Dextrose Agar (SDA) medium supplemented with chloramphenicol (0.4 µg/mL) to suppress bacterial growth. Explants were positioned equiangularly at an estimated center-to-center distance of 2–3 cm to avoid colony interference. Plates were incubated aerobically at 37˚C for 48 h. Emergent fungal colonies from the mesophyll tissue were purified through repeated subculturing. To ensure the recovery of both sporulating and non-sporulating (sterile) taxa, purification was performed using the hyphal tip technique via agar block transfer. Specifically, a 5x5 mm agar plug containing the actively growing mycelial front was excised from the primary culture and inoculated onto fresh SDA plates. This process was repeated until morphological uniformity was achieved, yielding pure axenic isolates.

## Phenetic characterization and cluster analysis

Phenetic characterization was performed on each purified isolate based on 33 macromorphological traits of colony morphologhy^19^, including both upper and reverse colony characteristics. Each character state (Table 1) was recorded in binary format (presence = 1, absence = 0) and compiled into a binary data matrix. Similarity analysis was conducted using the Jaccard coefficient via the UPGMA clustering tool available at http://genomes.urv.cat/UPGMA/. The resulting Newick-formatted (.nwk) dendrogram file was visualized in RStudio (2025.05.1–513.1) using the *ape* and *ggtree* packages to generate a phenetic dendrogram^20^. One representative isolate from each major cluster was selected for bioactivity screening, with priority given to isolates producing visible extracellular exudates.

**Table 1:** List of morphological characters and binary coding system utilized for the numerical taxonomic classification of red betel symbiotic fungi.

**No**	**Character category**	**Phenotypic trait description**	Variable code*	**Binary scoring**
1	Colony pigmentation (Upper surface)	White	WUC-P	1 = Present, 0 = Absent
2		Black/Dark	WUC-H	1 = Present, 0 = Absent
3		Orange	WUC-O	1 = Present, 0 = Absent
4		Purple	WUC-U	1 = Present, 0 = Absent
5	Colony pigmentation (Reverse surface)	White	WRC-P	1 = Present, 0 = Absent
6		Black/Dark	WRC-H	1 = Present, 0 = Absent
7		Orange	WRC-O	1 = Present, 0 = Absent
8		Purple	WRC-U	1 = Present, 0 = Absent
9	Surface texture	Velvety	TK-B	1 = Present, 0 = Absent
10		Smooth/Glabrous	TK-H	1 = Present, 0 = Absent
11		Powdery	TK-P	1 = Present, 0 = Absent
12		Cottony (Floccose)	TK-K	1 = Present, 0 = Absent
13		Rough	TK-S	1 = Present, 0 = Absent
14	Margin configuration	Entire (Smooth edge)	MG-E	1 = Present, 0 = Absent
15		Undulate (Wavy)	MG-U	1 = Present, 0 = Absent
16		Lobate (Lobed)	MG-L	1 = Present, 0 = Absent
17	Elevation	Flat	ELE-D	1 = Present, 0 = Absent
18		Convex	ELE-C	1 = Present, 0 = Absent
19		Raised (Umbonate)	ELE-J	1 = Present, 0 = Absent
20		Concave (Crateriform)	ELE-CK	1 = Present, 0 = Absent
21	Growth rate	Rapid (< 7 days to cover plate)	LJ-C	1 = Yes, 0 = No
22		Slow (> 7 days to cover plate)	LJ-L	1 = Yes, 0 = No
23	Metabolite production	Exudate/Pigment diffusion	PEP	1 = Present, 0 = Absent
24		Substrate clearing (Halo)	ZB	1 = Present, 0 = Absent
25	Consistency	Firm	KS-P	1 = Present, 0 = Absent
26		Mucilaginous/Slimy	KS-L	1 = Present, 0 = Absent
27		Dry	KS-K	1 = Present, 0 = Absent
28		Moist	KS-LB	1 = Present, 0 = Absent
29	Odor	Earthy	AM-T	1 = Present, 0 = Absent
30		Alcoholic	AM-A	1 = Present, 0 = Absent
31		Fermentative	AM-F	1 = Present, 0 = Absent
32	Special features	Multicolored pattern	MUC	1 = Present, 0 = Absent
33		Zonate (Concentric rings)	ZNT	1 = Present, 0 = Absent

*Explanation of abbreviated Codes: Codes represent the variable identifiers used in the binary data matrix. WUC: White Upper Colony; WRC: White Reverse Colony; TK: Texture (*Tekstur*); MG: Margin; ELE: Elevation; LJ: Growth Rate (*Laju Pertumbuhan*); PEP: Pigment/Exudate Production; ZB: Zone of Clearing (*Zona Bening*); KS: Consistency (*Konsistensi*); AM: Aroma/Odor; MUC: Multicolored; ZNT: Zonate. Binary state "1" indicates the trait is present in the isolate; "0" indicates the trait is absent.

## Preliminary bioactivity screening

Selected isolates were rejuvenated on SDA plates and subsequently inoculated into red rice (*Oryza sativa* var. andel abang) as a substrate for small-scale solid-state fermentation^21^. Fermentation was conducted under aerobic conditions for 28 days at ambient temperature. The fermented substrate was extracted by maceration in methanol (1:3 v/v) for three days with occasional agitation, followed by filtration. The filtrate was concentrated under reduced pressure, and crude extracts were subjected into two preliminary assays: Kirby–Bauer disk diffusion assay against cariogenic bacteria (*Streptococcus mutans* ATCC 25175) with 2% chlorhexidine as a positive control; chemical profiling by thin-layer chromatography (TLC) using silica gel F_254_ and octadecylsilyl (ODS) stationary phases under UV light (254 and 365 nm).

## Molecular identification

For molecular identification, genomic DNA was extracted using the Quick-DNA Magbead Plus Kit (Zymo Research, D4082) following the internal protocol. The internal transcribed spacer (ITS) region was amplified with MyTaq HS Red Mix, 2X (Bioline, BIO-25048), and PCR products were verified by using agarose gel electrophoresis. Bidirectional Sanger sequencing was performed via capillary electrophoresis, and raw sequence data were assembled and analyzed using an in-house bioinformatics pipeline. Sequencing data were used for BLAST searches against the NCBI GenBank database to determine species-level identity and infer phylogenetic relationships^22^.

## Results

### Sampling distribution

The distribution map illustrates the spatial variation of *Piper crocatum* (red betel) populations sampled across Tasikmalaya Regency, West Java (Figure S1, Table S1). A total of 13 accessions (SM1001–SM2013) were collected, representing diverse subdistricts ranging from lowland to upland areas. The sampling sites cover heterogeneous ecological zones, which are likely to influence the phytochemical diversity and bioactive metabolite profiles of the plants. Spatial heterogeneity is particularly important in medicinal plants, as environmental conditions such as altitude, soil composition, and microclimatic factors can modulate secondary metabolite biosynthesis. This variability provides a strong foundation for bioactivity-guided selection, as distinct populations may yield differential antimicrobial or antibiofilm potentials against oral pathogens. The broad geographic coverage also ensures that the sampling design captures both ecological and chemotypic diversity, thus increasing the reliability of subsequent biological and chemical analyses^23–25^.

## Phenotypic clustering as a dereplication strategy

Given the high morphological plasticity of fungi, we employed phenetic clustering primarily as a dereplication strategy to reduce sample redundancy rather than as a definitive predictor of chemotaxonomy. This approach allowed for the rational selection of representative isolates from a large pool. A total of 66 fungal isolates obtained from *Piper crocatum* (Table S2) exhibited substantial morphological diversity, as reflected by variations in colony pigmentation, texture, margin configuration, elevation, and growth rate (Table S1). Such heterogeneity is typical of mycobiont communities and underscores their underlying taxonomic and ecological diversity. Comparable morphological differentiation has been reported in mycobiont associated with other medicinal plants, reinforcing the notion that *P. crocatum* represents a rich reservoir of unexplored microbial taxa.

**Fig. 1:**
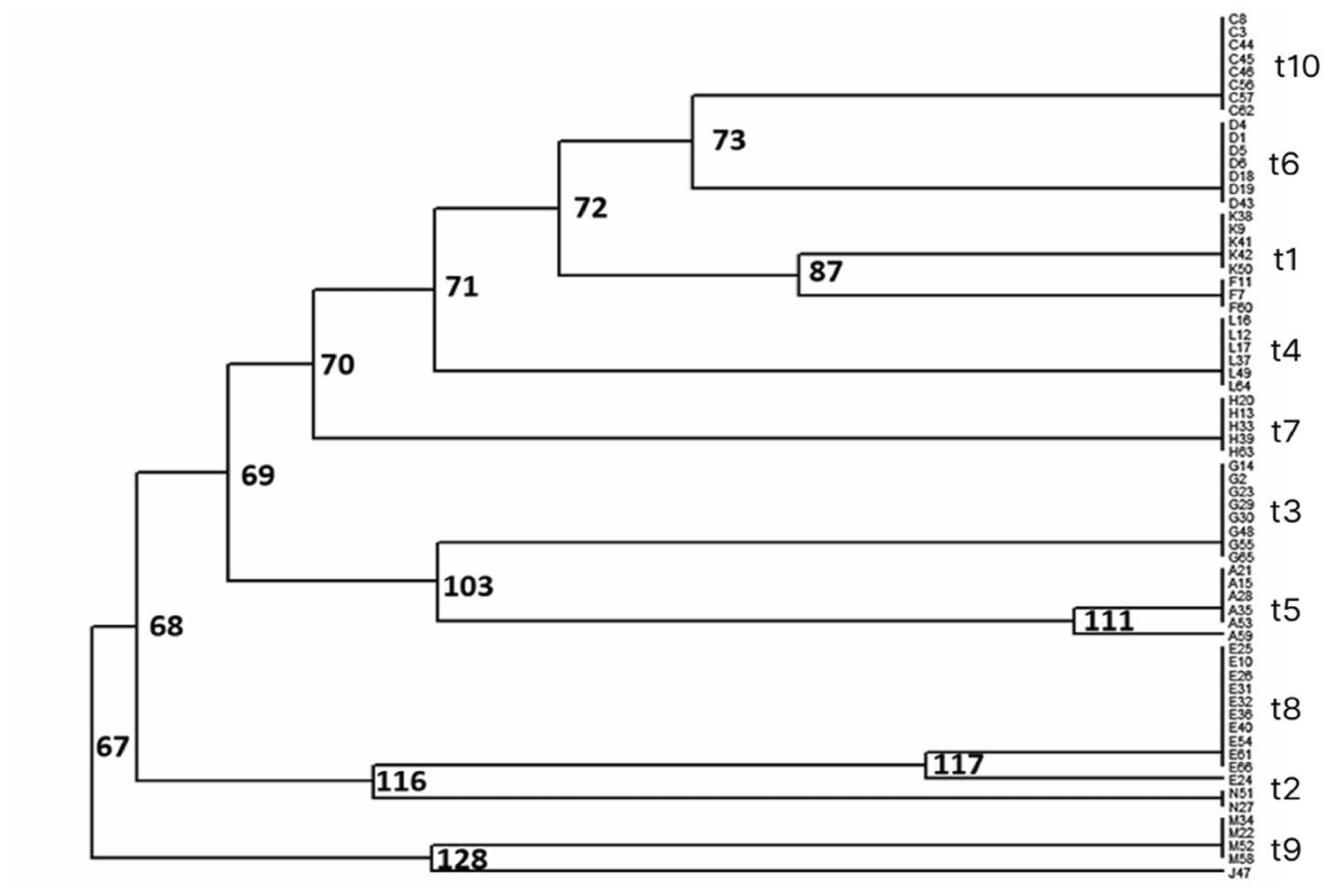
Hierarchical clustering dendrogram classifying 66 *Piper crocatum* mycosimbont fungal isolates into ten distinct phenotypic groups (t1–t10). The dendrogram was generated using the UPGMA method based on Jaccard similarity coefficients derived from 33 macroscopic morphological traits. Numerical values at the branching points indicate bootstrap support, reflecting the stability of the cluster topology. The ten major clusters (t1–t10) represent defined "morphotypes." Isolates within the same cluster share high phenotypic similarity. This classification served as a dereplication framework to select representative strains for downstream metabolite profiling and molecular identification.

To resolve relationships among isolates, 33 macromorphological traits (Table 1) were subjected to multivariate analyses. Cluster analysis using the UPGMA method (Figure 1) partitioned the isolates into several well-defined groups with moderate to high bootstrap support (70–128). The presence of strongly supported clades (bootstrap ≥100) suggests that certain colony features—particularly pigmentation, texture, and margin structure, can serve as indicators of taxonomic proximity, although other traits may be influenced by culture conditions^26^.

**Fig. 2:**
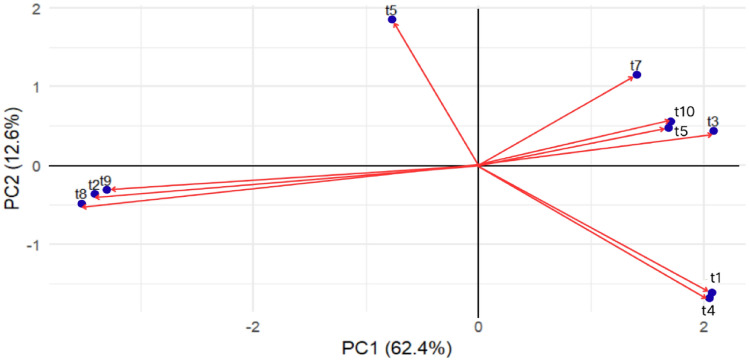
Principal Component Analysis (PCA) biplot characterizing the phenotypic diversity of *Piper crocatum* mycosimbiotic fungi. The plot visualizes the morphological variance among the ten isolate clusters (t1–t10) based on 33 binary traits. The first two principal components explain 75% of the total variance: PC1 (62.4%) primarily separates isolates by growth rate and pigmentation, while PC2 (12.6%) is associated with texture and elevation. (Interpretation) The spatial separation reveals distinct morphotypes. The clusters on the left (t2, t8, t9) represent the slow-growing, non-pigmented lineage, which is phenotypically distinct from the fast-growing, pigmented clusters distributed on the right (t1, t4 and t3, t7, t10). The length and direction of the red vectors indicate the magnitude of divergence of each cluster from the phenotypic mean.

**Fig. 3:**
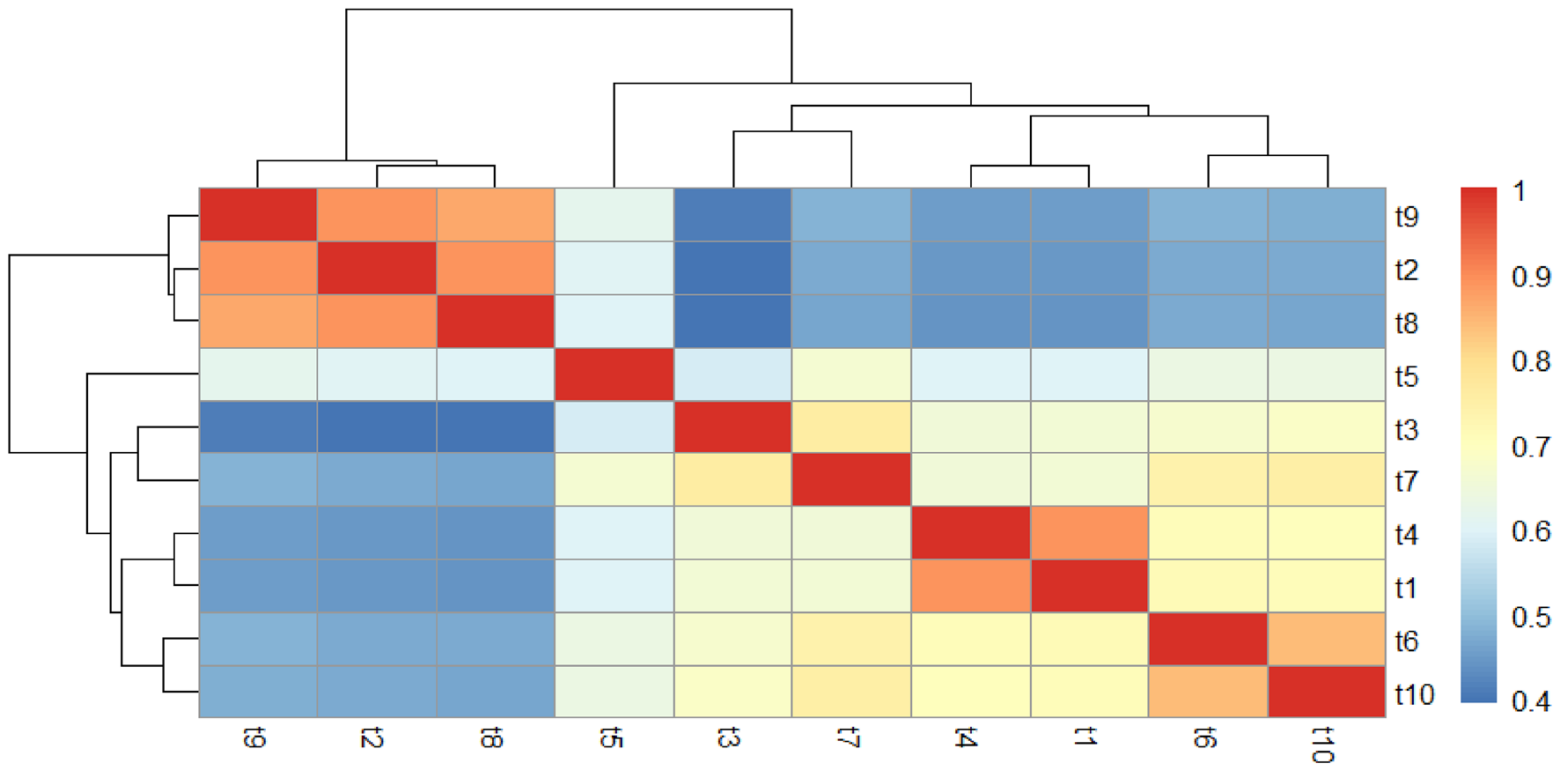
Phenotypic similarity matrix and hierarchical clustering of *Piper crocatum* mycosymbion. The heatmap displays pairwise Jaccard similarity coefficients derived from 33 macromorphological traits across the ten defined isolate clusters (t1–t10). The color gradient represents the degree of phenotypic overlap, ranging from blue (coefficient ~0.4, low similarity) to red (coefficient 1.0, high similarity). Dark red blocks along the diagonal indicate high intra-cluster homogeneity, confirming that isolates within groups share consistent morphological features.

Principal component analysis (PCA) clarified phenotypic variation, with the first two principal components (PC1 and PC2) explaining 75% of the cumulative variance (Figure 2). Separation along PC1 was primarily associated with colony color and growth rate, distinguishing fast-growing, pigmented clusters (t1, t3, t4, t10) from slower-growing, less-pigmented clusters (t2, t8, t9) (Figure 3). In contrast, PC2 was largely driven by differences in texture and elevation, separating clusters such as t5 and t7 that displayed unique colony surface structures. These results indicate that distinct morphological traits, particularly pigmentation and texture, can serve as reliable markers for preliminary classification prior to molecular identification.

Complementary insights were obtained from a Jaccard similarity heatmap (Figure 3), which summarized binary trait overlap across clusters (t1–t10). Similarity coefficients ranged from 0.40 to 1.00, revealing both highly conserved groups and divergent profiles. Cluster A (t9, t2, t8) displayed the highest within-group similarity (>0.85), whereas Cluster B (t3, t7, t4, t1) exhibited moderate similarity (0.75–0.85). Cluster C (t6 and t10) demonstrated an especially close relationship (~0.90), while isolate t5 emerged as an intermediate, bridging distinct groups. This clustering pattern suggests that while subsets of isolates share conserved morphologies, others may represent ecologically or functionally divergent taxa.

Isolates from geographically distant sites were distributed across multiple clusters. While environmental factors are known to significantly shape mycosymbiont communities, which in turn can modulate the host’s chemical profile^27^, the recurrence of specific fungal taxa across diverse sampling locations in this study suggests a degree of host specificity or ‘host filtering.’ This implies that *P. crocatum* may select for a core set of mycosymbiont associates from the environmental pool, potentially recruiting compatible partners that can thrive within its specific internal chemical milieu. Capitalizing on this ecological structuring, the integration of morphological characterization, UPGMA clustering, PCA, and Jaccard similarity mapping provides a rational framework for selecting representative isolates. By prioritizing morphologically distinct and phylogenetically robust clusters, the likelihood of capturing metabolite diversity is maximized, thereby enhancing the efficiency of bioactivity-guided screening for novel antimicrobial compounds.

**Fig. 4:**
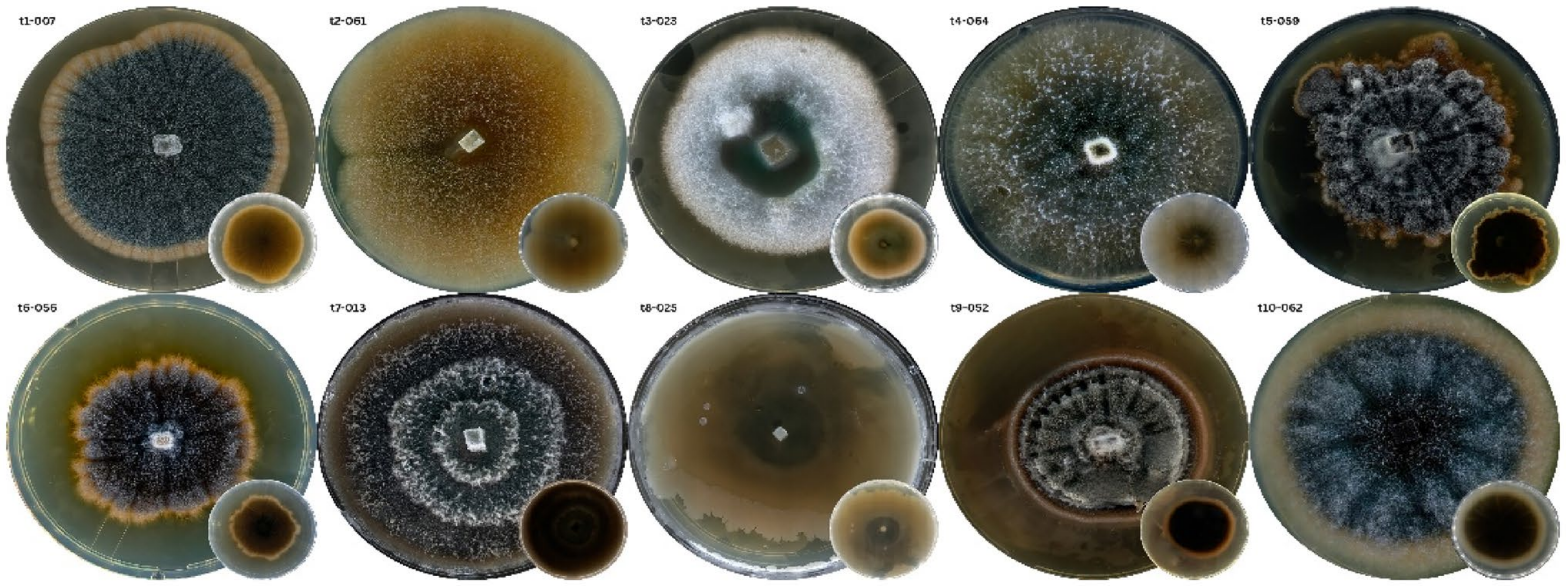
Representative fungal colonies corresponding to clusters (t1-t10) in the Jaccard-based phenogram.

**Fig. 5:**
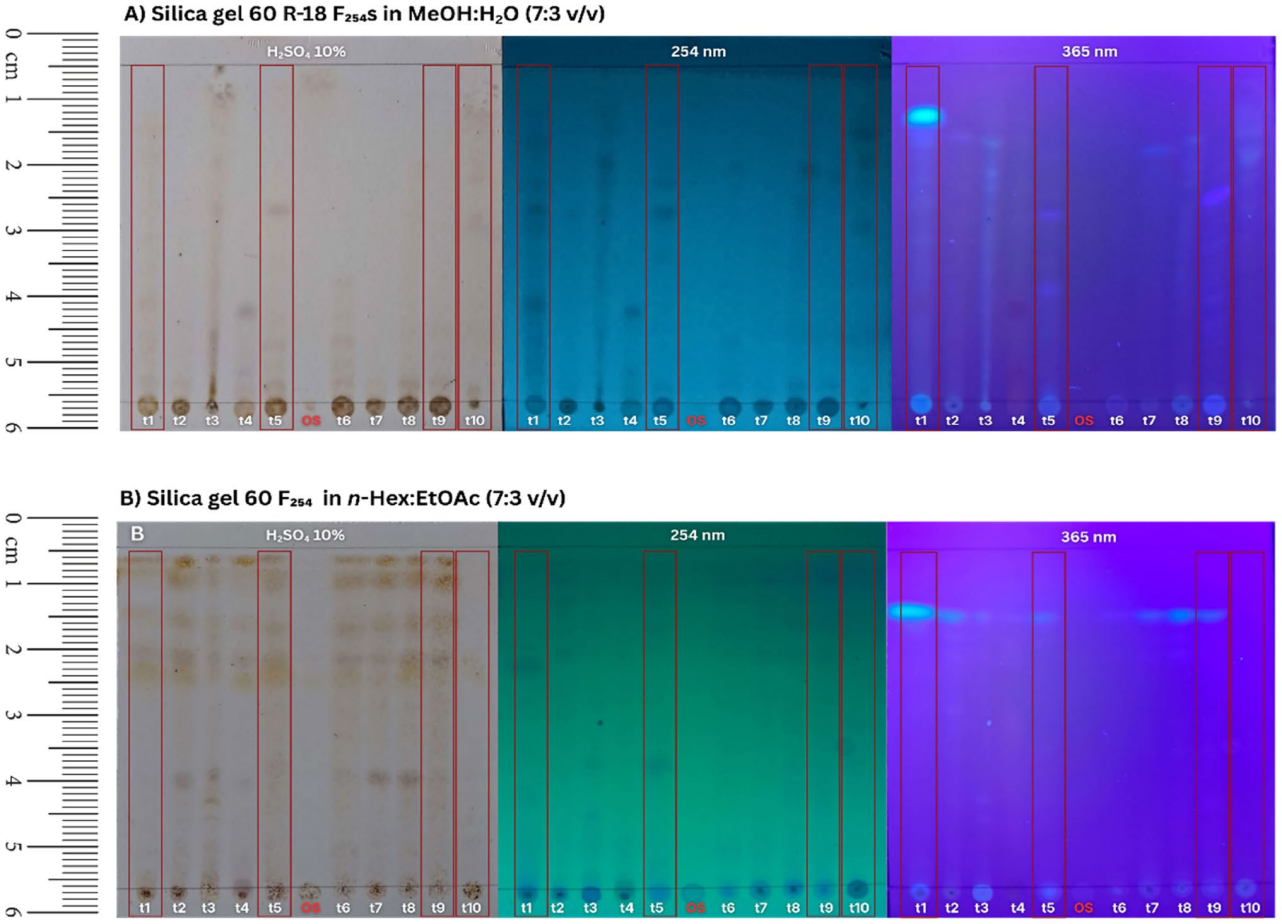
Comparative Thin-Layer Chromatography (TLC) profiles of methanol extracts from ten mycosimbiotic fungal isolates and the unfermented host substrate. Chromatograms were developed on (**A**) silica gel 60 R-18 F₂₅₄s plates in methanol:distilled water (7:3 v/v) and (**B**) silica gel 60 F₂₅₄ s in *n*-hexane:ethyl acetate (7:3 v/v) as mobile phases. Visualization was performed using 10% H_2_SO_4_ spray reagent followed by heating, UV light at 254 nm and 365 nm. Lanes 01–10: Extracts from mycosimbiotic fungal fermentation (t1–t10). Lane OS: Extract from sterile, unfermented red rice (*Oryza sativa* var. *andel abang*) substrate, included as a negative control to differentiate fungal secondary metabolites from substrate-derived constituents.

While morphological traits do not always correlate with secondary metabolite production, our PCA and TLC analyses (Figure 3, Figure 4, Figure 5) revealed a discernable pattern: clusters defined by specific pigmentation and growth rates corresponded to isolates with higher metabolic complexity and stronger antimicrobial activity. This suggests that, within the context of this specific community, phenotypic differentiation served as a useful, albeit low-resolution, proxy for functional diversity.

## Preliminary metabolite profiling and antimicrobial screening of representative mycosymbiotic fungi

One representative strain from each major cluster (Figure 4) was selected for preliminary metabolite screening. This strategy ensured that the functional potential of phylogenetically and morphologically distinct taxa could be captured, thereby increasing the likelihood of identifying novel bioactive metabolites. Ten representative isolates from each cluster (sample code: t1-007, t2-061, t3-023, t4-064, t5-059, t6-056, t7-013, t8-025, t9-052, t10-062) were fermented aerobically on a small scale using sterile red rice as substrate, providing a nutrient-rich and reproducible matrix for metabolite production.

**Fig. 6:**
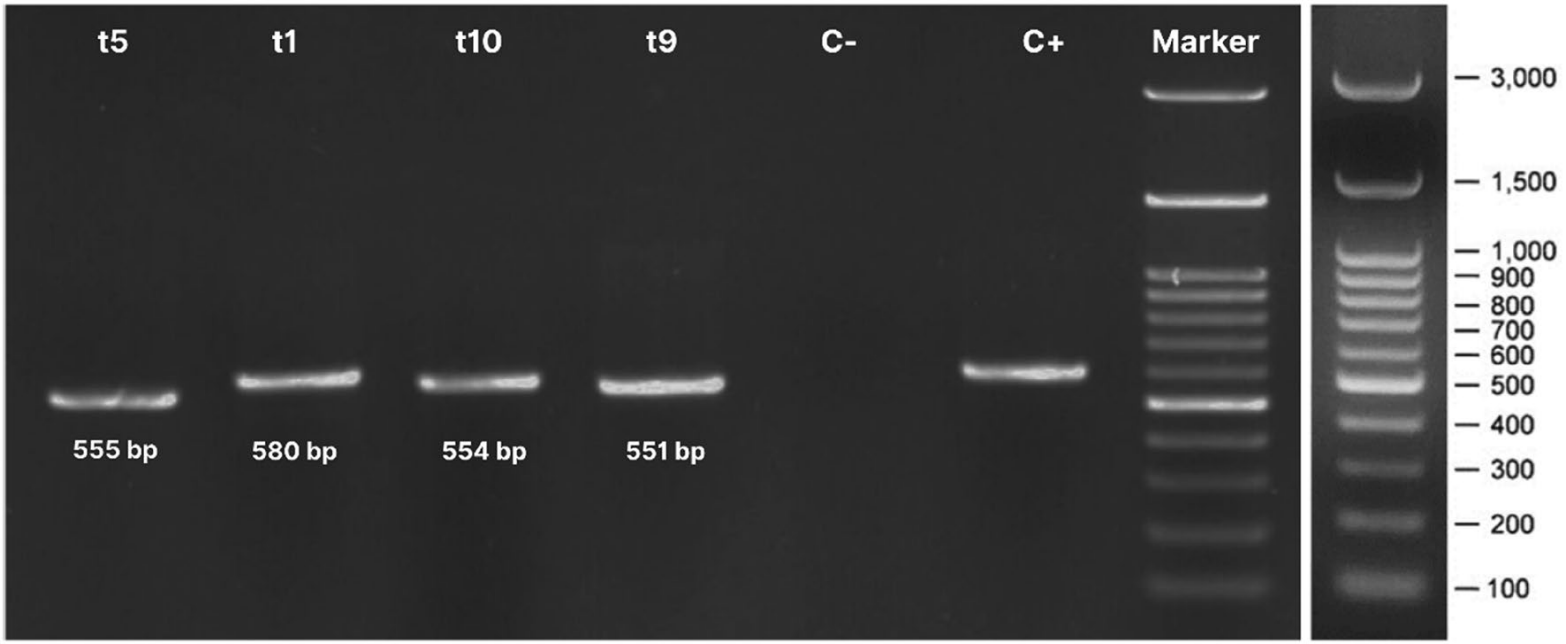
Agarose gel electrophoresis of PCR amplicons targeting the ITS rDNA region of representative fungal morphotypes. Lanes t5, t1, t10, t9 PCR products amplified from representative isolates belonging to morphological clusters, respectively. The distinct bands (551–580 bp) confirm the amplification of the complete ITS1-5.8S-ITS2 region. C-: Negative control (PCR mixture without template DNA) showing no contamination. C+: Positive control (fungal genomic DNA) confirming reaction viability. 100 bp DNA ladder (100–3,000 bp) used for size estimation.

After 14 days of incubation, the fermented material was extracted by methanolic maceration, subjected to sonication-assisted lysis, and concentrated using rotary evaporation to obtain crude extracts. Thin-layer chromatography (TLC) analysis revealed marked differences in metabolite banding patterns among the isolates, highlighting the chemical heterogeneity of the mycosymbiont community. Distinct UV-active spots were observed at both 254 nm and 365 nm, with some extracts displaying unique fluorescent bands (Figure 6). The appearance of isolate-specific chromatographic fingerprints supports the notion that each cluster contributes different metabolic capacities, consistent with previous studies demonstrating that fungal mycosymbiont from medicinal plants produce diverse classes of secondary metabolites, including alkaloids, terpenoids, and polyketides^28^.

The comparative TLC profiling reveals distinct chemical differences between the active fungal isolates (t1, t5, t9, t10) and the inactive ones. On normal-phase silica gel (Figure 5B), the active extracts display prominent bands with high Rf values, which correspond to retained bands with low Rf values on the reverse-phase plates (Figure 5A). This migration pattern indicates that the potentially bioactive secondary metabolites are predominantly non-polar to semi-polar in nature. Visualization under UV light, particularly at 365 nm, reveals intense blue fluorescent bands in these specific lanes that are notably absent in the unfermented substrate control (OS), confirming these compounds are unique products of fungal fermentation rather than substrate constituents.

There is a strong positive correlation between the intensity of these fluorescent TLC bands and the anticariogenic activity recorded against *S. mutans* (Table 2)*.* Isolate t5, which exhibited the highest inhibition zone (21.50 mm), corresponds to the lane with the most intense and distinct fluorescent bands in the chromatogram. Following this pattern, isolates t9 (15.85 mm), t10 (12.75 mm), and t1 (11.50 mm) show progressively weaker fluorescence, mirroring their decreasing bioactivity. Conversely, isolates with minimal inhibition zones (t2, t3, t4, t6) lack these characteristic metabolite bands entirely, displaying profiles similar to the inactive negative control.

**Table 2:** Inhibition zone of mycosimbiotic fungal extracts for anticariogenic activity against *S. mutans* via agar disc diffusion assay (mm).

**Sample**	Inhibition zone (mm)*		$\rm{\overline{X}}$**(mm) ± Dev. standard**
t1	12.0	11.0	11.50 ± 0,50
t2	6.0	5.0	5.50 ± 0,50
t3	5.0	5.0	5.00 ± 0
t4	5.0	6.0	5.50 ± 0.50
t5	21.0	22.0	21.50 ± 0.50
t6	5.0	5.0	5.00 ± 0
t7	7.0	6.8	6.90 ± 0.10
t8	6.5	6.0	6.25 ± 0.25
t9	16.0	15.7	15.85 ± 0.15
t10	13.0	12.5	12.75 ± 0.25
**C(+) Chx 2%	17.0	17.0	17.00 ± 0
**C(-) MeOH	5.0	5.0	5.00 ± 0

*diameter of the paper disc: 5 mm.

**Positive control using chlorhexidine 2%.

**Negative control using methanol.

In conclusion, the specific non-polar, blue-fluorescent metabolites identified in the TLC profiles serve as reliable chemical markers for the observed anticariogenic potency. The fluorescence at 365 nm and quenching at 254 nm suggest the active compounds possess conjugated systems, likely aromatic structures such as phenolics or coumarins. Consequently, the TLC fingerprint confirms that isolate t5 is the most prolific producer of these bioactive metabolites, making it the priority candidate for subsequent compound isolation and structural elucidation.

When these results were integrated with the TLC metabolite fingerprints, a compelling pattern emerged. Isolates demonstrating strong antimicrobial activity (t1-007, t5-059, t9-052, and t10-062) also exhibited richer chromatographic profiles, characterized by multiple UV-active bands under both 254 nm and 365 nm illumination. In particular, isolate t5-059 displayed several unique fluorescent spots, suggesting the presence of diverse and potentially bioactive secondary metabolites. This correlation between chemical complexity and bioactivity supports the use of TLC as a rapid, cost-effective proxy for identifying metabolically prolific isolates. The most potent isolate (t5-059), originated from a cluster defined by slow-growing, pigmented colonies, further reinforcing the notion that pigmentation is linked with metabolite production in mycosymbiotic fungi. The alignment of morphological traits, metabolite diversity, and strong bioactivity highlights the robustness of the integrated selection pipeline employed in this study.

## Molecular identification of selected fungal isolate

**Fig. 7:**
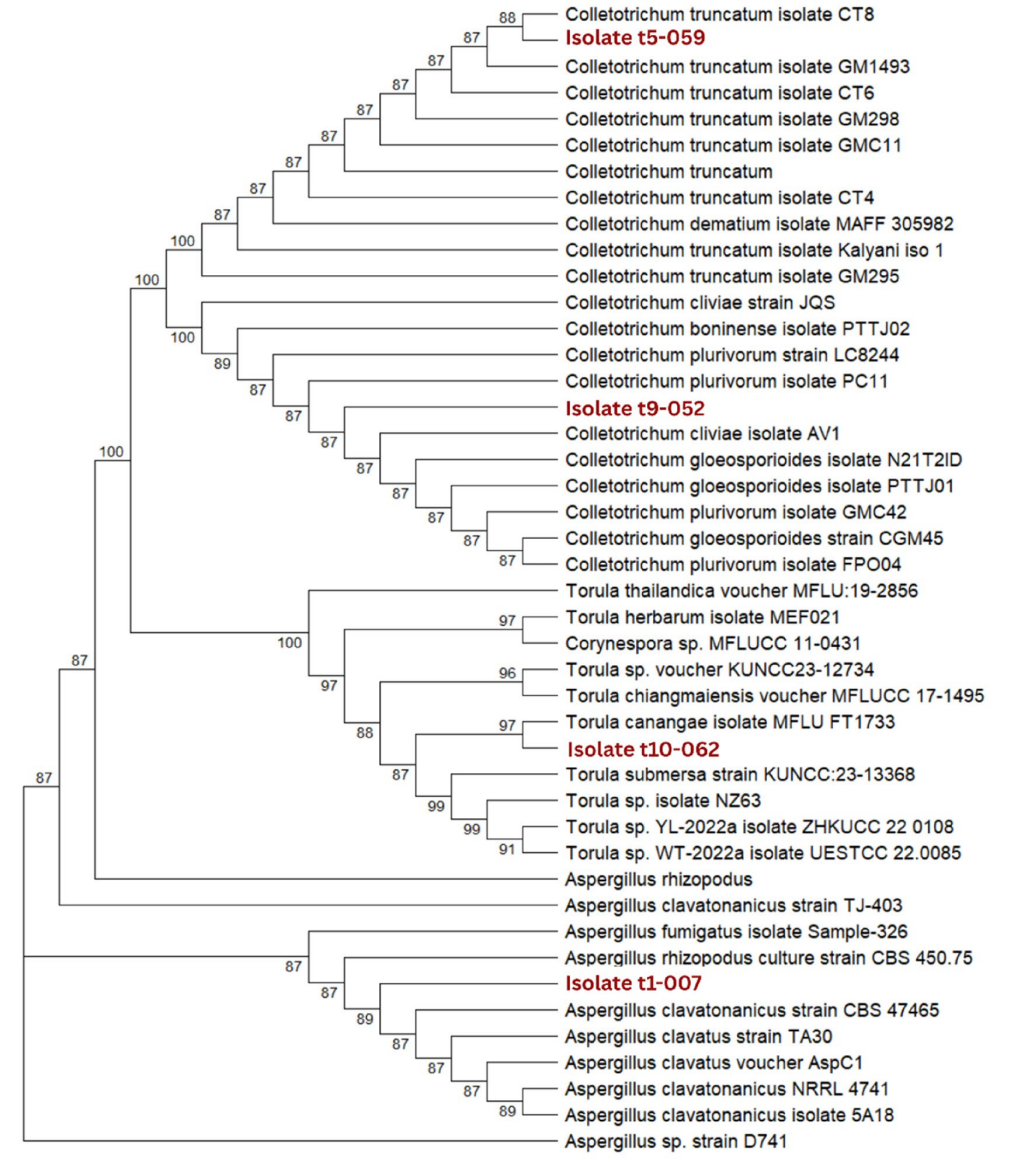
Molecular phylogeny of mycosimbionts from *Piper crocatum* based on ITS rDNA sequences reveals distinct taxonomic clusters. The phylogenetic tree was reconstructed using the Internal Transcribed Spacer (ITS1-5.8S-ITS2) region sequences. The analysis places the Piper crocatum-associated endophytes into three distinct generic clades: *Colletotrichum, Torula*, and *Aspergillus*. (Isolates) Taxa highlighted in red represent the bioactive isolates obtained in this study. The labels follow the format "Cluster-Isolate Code" (t5-059 indicates isolate 059 belonging to Morphological Cluster t5), ensuring traceability to the phenotypic data. Numbers at the nodes indicate bootstrap support values (%) based on 1,000 replicates; values 50% are omitted. The analysis confirms the identity of Isolate t5-059 as *Colletotrichum truncatum*, isolate t10-062 as *Colletotrichum cliviae*, isolate t9-052 as *Torula canangae*, and isolate t1-007 as *Aspergillus clavatonanicus*. Reference sequences were retrieved from the NCBI GenBank database (Table S3).

Molecular characterization of the four representative isolates (t1-007, t5-059, t9-052, t10-062) using the ITS rDNA region confirmed their taxonomic affiliation with diverse fungal genera. PCR amplification produced clear amplicons within the expected size range (Figure 6), which were subsequently sequenced and compared against 40 reference strains retrieved from NCBI. Phylogenetic analysis demonstrated robust clustering of the isolates with *Colletotrichum*, *Torula*, and *Aspergillus* lineages, supported by high bootstrap values (Figure 7). Specifically, isolate t5-059 grouped with *Colletotrichum truncatum*, isolate t9-052 with *Colletotrichum cliviae*, isolate t10-062 with *Torula canangae*, and isolate t1-007 with *Aspergillus clavatonanicus*.

These results provide novel insight into the mycosymbiotic mycobiota of *Piper crocatum*, a medicinal plant that has not previously been extensively surveyed for fungal associates. While *Colletotrichum* species are widely recognized as phytopathogens, their role as endophytes producing bioactive metabolites has only recently gained attention. Similarly, *Torula*, traditionally understudied in endophyte research, has been increasingly reported as a promising source of antimicrobial and antibiofilm compounds. The recovery of *Aspergillus clavatonanicus*, a rare species with limited documentation in mycosymbiotic contexts, further expands the taxonomic spectrum of fungi associated with red betel.

Comparatively, most previous reports of endophytes from medicinal *Piper* species have been dominated by *Fusarium*, *Penicillium*, and *Aspergillus* spp., whereas the detection of *T. canangae* and *C. cliviae* in this study represents, to our knowledge, the first report of these taxa as mycosimbiont in red betel. This finding highlights the unique ecological niche of red betel that accommodates both pathogenic and rare fungal taxa, suggesting an evolutionary adaptation that may stimulate the biosynthesis of structurally diverse secondary metabolites. The novelty of this study lies not only in uncovering unexpected fungal diversity withinn red betel but also in identifying endophytes with previously unreported associations that hold significant potential for the discovery of anticariogenic compounds. Given the established antimicrobial and quorum-sensing inhibitory properties of *Colletotrichum* and *Aspergillus* metabolites, and the emerging relevance of *Torula* spp., these isolates represent promising candidates for further exploration in dental and oral health applications.

## Discussion

### Fungal diversity and methodological efficacy

The recovery of 66 isolates from red betel leaves across heterogeneous sampling sites reveals a diverse fungal community inhabiting the mesophyll tissues. The high morphological variation observed, ranging from pigmented to sterile mycelia, aligns with the concept that medicinal plants harbor complex microbiomes driven by unique host chemical niches. The application of numerical taxonomy based on 33 phenotypic traits proved effective in resolving these isolates into ten major clusters^29^. Notably, the grouping of isolates from geographically distant locations into shared phenotypic clusters supports the hypothesis of host-driven selection, or "host filtering," where red betel recruits specific functional guilds regardless of the surrounding environment.

### Correlation between chemical profiling and bioactivity

A key finding of this study is the positive correlation between metabolite complexity and anticariogenic activity. The TLC profiling revealed that isolates belonging to pigmented, slow-growing clusters (*C. truncatum* isolate t5-059) produced a higher diversity of secondary metabolites. This chemical richness corresponded directly with biological efficacy; exhibited the strongest inhibition against *S. mutans* (21.5 mm). This observation supports the "defense-by-pigment" hypothesis, according to Afroz Toma et al.^30^ where fungal pigments (often polyketides or quinones) serve dual roles as UV protectants and antimicrobial agents. The distinct fluorescent bands observed under UV 365 nm for this sample suggest the presence of conjugated systems typical of bioactive alkaloids or phenolics, warranting future structural elucidation.

### Taxonomic context and ecological lifestyle

Molecular identification revealed taxa that are traditionally viewed as phytopathogens or saprotrophs, specifically *C. truncatum* and *C. cliviae*. The isolation of these species from healthy, asymptomatic red betel tissues highlights the complex “lifestyle switching” of fungal endophytes. *Colletotrichum* species are known to exist as latent pathogens (quiescent endophytes) before switching to a pathogenic phase, a strategy that likely involves the secretion of antimicrobial metabolites to suppress competing microbes within the host. These finding of *Colletotrichum* species from asymptomatic red betel tissues is best explained by the concept of hemibiotrophy, a sophisticated nutritional strategy characterized by a temporal lifestyle switch^30–32^. Unlike strict necrotrophs that immediately kill host tissue, these taxa initiate infection with a distinct biotrophic or quiescent phase, during which they colonize the intercellular spaces (apoplast) without causing cellular damage or provoking a hypersensitive immune response. In this “mycosymbiotic” stage, the fungus acts as a latent colonizer, often suppressing its own virulence factors and secreting specific effector proteins to evade host detection^33^. Therefore, recovering these fungi from healthy surface-sterilized leaves does not imply contamination; rather, it captures them during this stable, asymptomatic period where they function ecologically as endophytes, awaiting specific cues to transition into pathogenicity^34^.

The transition from this quiescent mycosymbiotic state to a necrotrophic (pathogenic) lifestyle is typically triggered by host senescence, physiological stress, or a decrease in plant immune signaling^35^. From an evolutionary perspective, the strong antimicrobial activity observed in isolates against *S. mutans* may be a functional trait of this competitive lifestyle. During the latent phase, these “waiting” pathogens likely secrete secondary metabolites not to kill the plant, but to exclude microbial competitors from the host niche—effectively guarding their food source until the host weakens. Consequently, the presence of *C. truncatum* and *C. cliviae* in this study highlights the plasticity of fungal symbiosis, where the distinction between a beneficial endophyte and a latent pathogen is not determined by the organism’s identity, but by the physiological status of the host and the timing of the interaction.

### Addressing ecological status, lifestyle switching novelty, and potential colonization

The identification of *T. canangae* and *A. clavatonanicus* represents an expansion of the known mycosymbiont associated with *Piper* species. While the rigorous surface sterilization protocol employed (sequential ethanol and NaOCl immersion) supports their status as internal colonizers, we interpret these findings with caution. These taxa may represent facultative endophytes or opportunistic colonizers recruited from the environment rather than obligate symbionts. Unlike the specialized *Colletotrichum* strains, *A. clavatonanicus* is rarely reported as an endophyte, suggesting its presence may be transient. The isolation of *Colletotrichum* species, traditionally characterized as phytopathogens, from healthy red betel leaves raises important questions regarding their ecological status. In this study, we adopted the operational definition of endophytes as microorganisms colonizing internal plant tissues without causing apparent disease symptoms at the time of sampling. We acknowledge that many endophytes, particularly *Colletotrichum* spp., possess hemibiotrophic lifestyles, capable of switching from a quiescent mycosymbiotic phase to a pathogenic phase depending on host senescence or environmental stress. Therefore, while these isolates were strictly mycosymbiotic at the time of isolation, they may represent latent pathogens or "facultative endophytes”. However, its production of bioactive metabolites against oral pathogens implies that even transient associates may contribute to the plant’s defensive chemical arsenal^36,37^. Future pathogenicity assays (Koch’s postulates) and histological tracking are necessary to definitively map the continuum of their interaction with red betel.

### Clinical implications

The identification of potent anticariogenic activity in *C. truncatum*, *C. cliviae, T. canangae*, and *A. clavatonanicus* carries significant clinical implications for the management of dental caries, particularly in high-prevalence regions like Indonesia. *Streptococcus mutans*, the primary etiological agent of caries, is notorious for forming recalcitrant biofilms that resist conventional antimicrobial penetration and contribute to treatment failure. Current standard-of-care agents, such as chlorhexidine and fluoride, face limitations including tooth staining, altered taste sensation, and the emergence of resistant bacterial phenotypes. The fungal endophytes isolated in this study, particularly *C. truncatum* (t5-059), which exhibited a mean inhibition zone of 21.5 mm, represent a promising reservoir of novel bioactive scaffolds that could circumvent these resistance mechanisms. Unlike broad-spectrum antibiotics that disrupt the entire oral commensal flora, natural products derived from these specific fungal symbiont may offer a more targeted approach to microbial modulation. The strong correlation observed between pigment production and bioactivity in these isolates suggests the presence of polyketide-based secondary metabolites, a chemical class with established efficacy in disrupting bacterial quorum sensing and biofilm architecture. Moving forward, the translation of these findings into clinical practice will require the isolation and structural elucidation of the active constituents, followed by rigorous cytotoxicity screening against human gingival fibroblasts to ensure safety. If validated in ex vivo biofilm models, these endophyte-derived metabolites could be developed as bioactive ingredients in next-generation oral healthcare products, such as therapeutic mouthrinses or slow-release dental varnishes, shifting the paradigm from restorative treatment to proactive ecological management of the oral microbiome.

## Conclusion

This study establishes the first comprehensive characterization of the mycosimbiont associated with red betel leaf, revealing a distinct community structure shaped by host-driven selection. By employing an inclusive isolation strategy utilizing hyphal tip transfer and a phenetic dereplication framework, we successfully recovered and prioritized diverse fungal taxa, including non-sporulating morphotypes often overlooked in conventional surveys. The recurrence of specific genera across geographically heterogeneous sites supports the hypothesis of "host filtering," suggesting that red betel recruits a core microbiome capable of thriving within its specific chemical niche. Notably, a strong correlation was observed between colony pigmentation and anticariogenic efficacy, with *C. truncatum* exhibiting the most potent inhibition against *Streptococcus mutans.* Molecular identification further confirmed *C. cliviae*, *T. canangae*, and *A. clavatonanicus* as key bioactive associates. Collectively, these findings position red betel-associated endophytes as a promising, renewable reservoir of bioactive scaffolds. Future efforts should focus on the bioactivity-guided fractionation of these crude metabolites and rigorous toxicity profiling to advance their development as effective, safe therapeutics for oral infectious diseases.

## Supplementary Information

Supplementary Information 1.

Supplementary Information 2.

## Data Availability

The nucleotide sequence data generated in this study have been deposited in the International Nucleotide Sequence Database Collaboration (INSDC) through GenBank (NCBI Nucleotide) under the following accession numbers: Colletotrichum truncatum strain SM_059_124A (PX415253), Aspergillus clavatonanicus strain SM_007_012 (PX415421), Colletotrichum cliviae strain SM_062_123 (PX415427), and Torula canangae strain SM_052_118 (PX415440). All sequences correspond to the ITS (Internal Transcribed Spacer) region and are publicly available at https://www.ncbi.nlm.nih.gov/nuccore. All other data generated or analysed during the current study are included in this published article and its Supplementary Information files.

## Materials availability

The fungal isolates obtained and characterized in this study are available from the corresponding author upon reasonable request.
