# Supplementary material for: Characterization of anticariogenic mycosymbiotic fungi associated with the medicinal plant Piper crocatum
Source: Sci Rep. 2026 Mar 18;16:13993. doi: 10.1038/s41598-026-41703-z (PMC13133259; doi:10.1038/s41598-026-41703-z)
Supplement: Supplementary file 1 — Supplementary Information 1. [file 41598_2026_41703_MOESM1_ESM.docx]

**Supplementary Material 1**

**Table S1** Material Samples of *Piper crocatum* locations across Tasikmalaya Regency

| **Code** | **Scientific Name** | **Coordinate** | **Specimen Source** |
| --- | --- | --- | --- |
| SM1001 | *Piper crocatum* | 7°20'18.8"S 108°14'22.6"E  -7.338567, 108.239608 | Cicurug, Tasikmalaya |
| SM1002 |  | 7°21'16.0"S 108°18'24.9"E  -7.354436, 108.306927 | Manonjaya, Tasikmalaya |
| SM1003 |  | 7°18'29.6"S 108°07'34.5"E  -7.308228, 108.126247 | Padakembang, Tasikmalaya |
| SM1004 |  | 7°17'28.7"S 108°07'59.8"E  -7.291294, 108.133286 | Sukaratu, Tasikmalaya |
| SM1005 |  | 7°21'02.8"S 108°06'47.5"E  -7.350768, 108.113206 | Singaparna, Tasikmalaya |
| SM1006 |  | 7°20'18.8"S 108°14'22.6"E  -7.338567, 108.239608 | Lanud, Tasikmalaya |
| SM2007 |  | 7°30'21.4"S 108°11'20.7"E  -7.505937, 108.189081 | Singajaya, Tasikmalaya |
| SM2008 |  | 7°44'24.6"S 108°13'13.3"E  -7.740164, 108.220356 | Kubangsari, Tasikmalaya |
| SM2009 |  | 7°20'47.6"S 108°14'00.4"E  -7.346563, 108.233439 | Setiaratu, Tasikmalaya |
| SM2010 |  | 7°20'54.4"S 108°14'03.8"E  -7.348431, 108.234385 | Cibeureum, Tasikmalaya |
| SM2011 |  | 7°18'35.5"S 108°13'31.7"E  -7.309856, 108.225479 | Cisalak, Tasikmalaya |
| SM2012 |  | 7°46'17.1"S 108°10'29.1"E  -7.771429, 108.174755 | Mandalajaya, Tasikmalaya |
| SM2013 |  | 7°21'43.4"S 108°13'12.8"E  -7.362043, 108.220222 | Tamansari, Tasikmalaya |


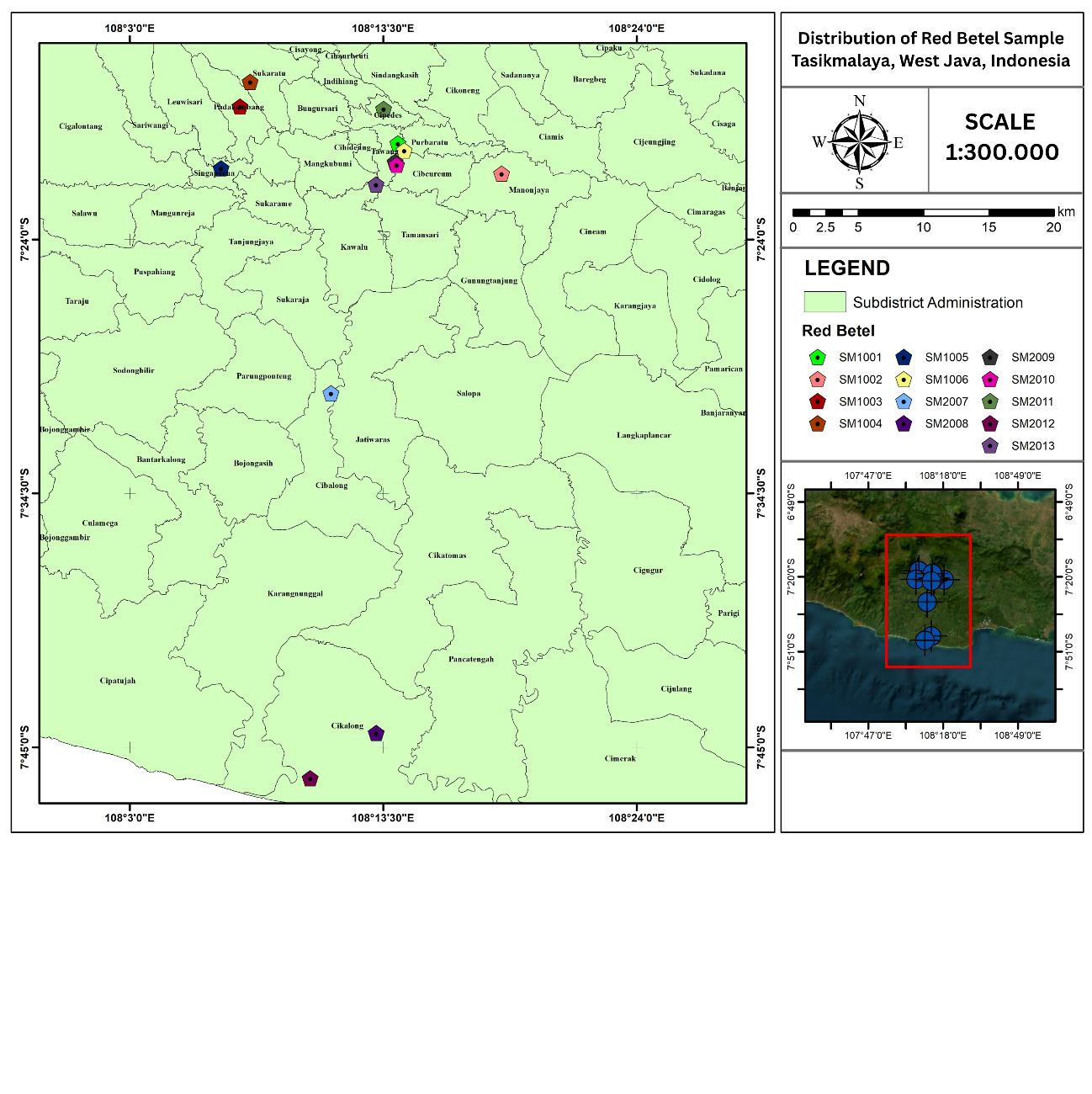


**Figure S1** Map showing the distribution of *Piper crocatum* sampling locations across Tasikmalaya Regency, West Java, Indonesia. The map was generated by the authors using ArcGIS Desktop version 10.8 (Esri, Redlands, CA, USA; <https://www.esri.com/>). Administrative boundary layers (province, district, and subdistrict) were obtained from the Rupa Bumi Indonesia (RBI) dataset provided freely by the Geospatial Information Agency of Indonesia (Badan Informasi Geospasial, BIG; <https://tanahair.indonesia.go.id/>). Sampling points (SM1001–SM1013) were plotted based on GPS coordinates recorded during fieldwork.

**Table S2** Geographic origin of red betel host accessions and morphological documentation of recovered fungal isolates

| **No** | **Code** | **Location** | **Leaf Picture** | **Saboraud Dextrose Agar (SDA) + chloramphenicol (0.4 µg/mL) Duplicated Innoculation** | | | |
| --- | --- | --- | --- | --- | --- | --- | --- |
|  |  |  |  | **Upper** | **Reverse** | **Upper** | **Reverse** |
| 1 | SM1001 | Cicurug | 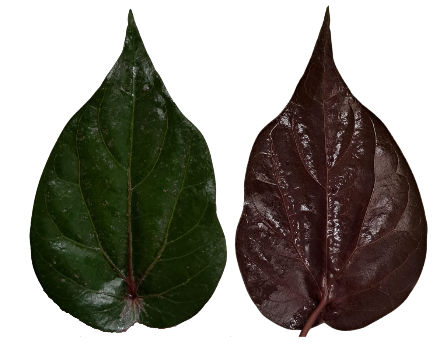 | 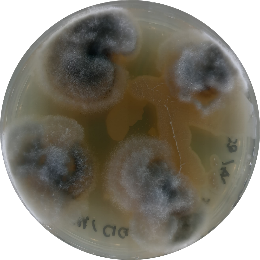 | 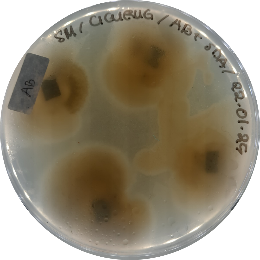 | 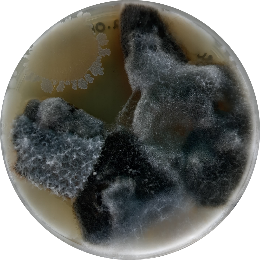 | 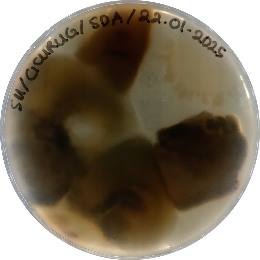 |
|  |  |  |  | **Reinoculated culture – recovered isolates**  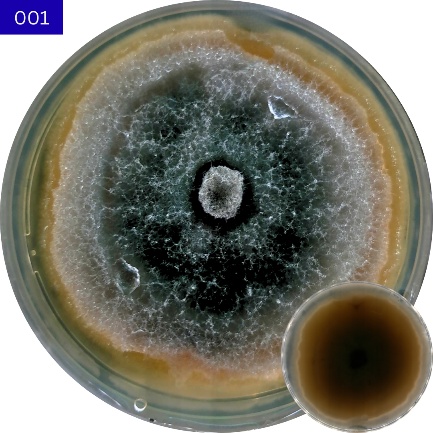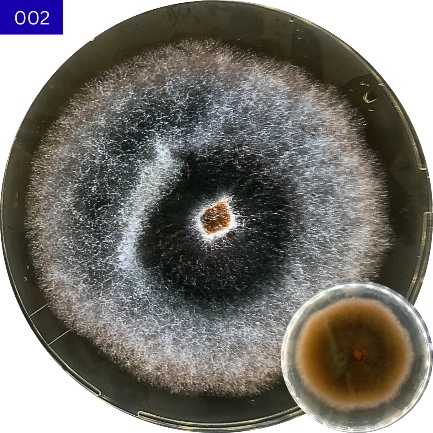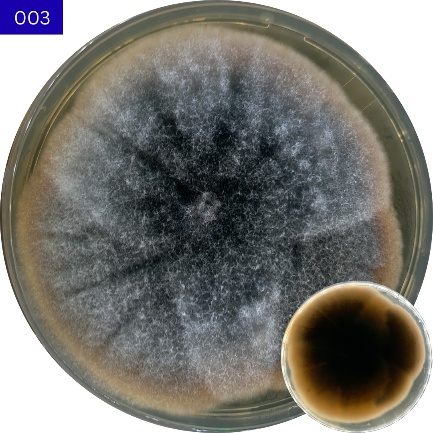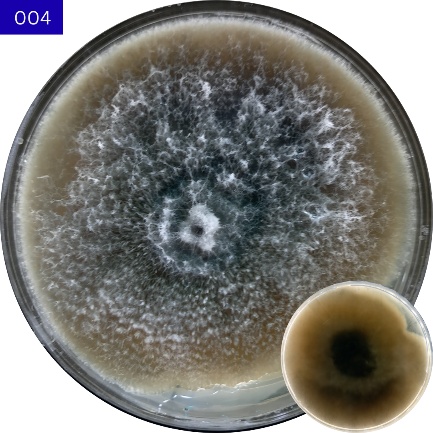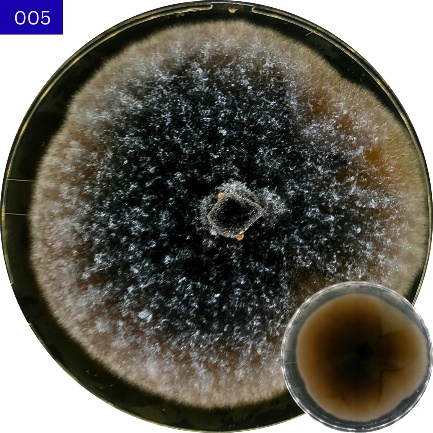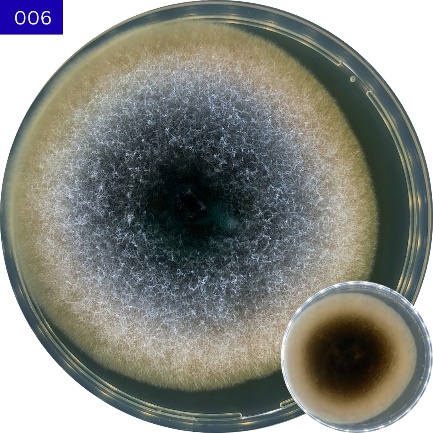 | | | |

| **No** | **Code** | **Location** | **Leaf Picture** | **Saboraud Dextrose Agar (SDA) + chloramphenicol (0.4 µg/mL) Duplicated Innoculation** | | | |
| --- | --- | --- | --- | --- | --- | --- | --- |
|  |  |  |  | **Upper** | **Reverse** | **Upper** | **Reverse** |
| 2 | SM1002 | Manonjaya | 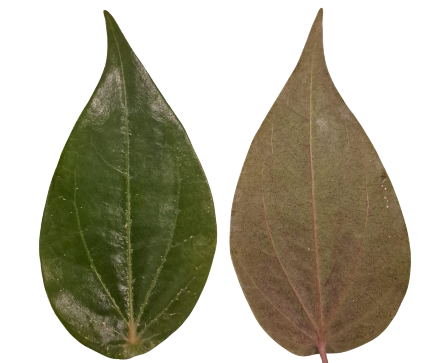 | 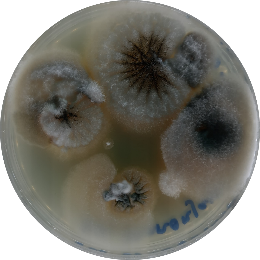 | 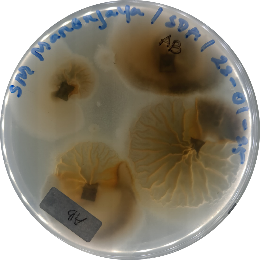 | 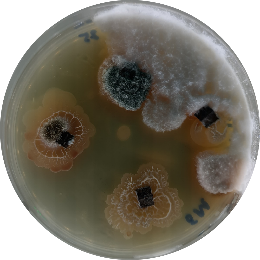 | 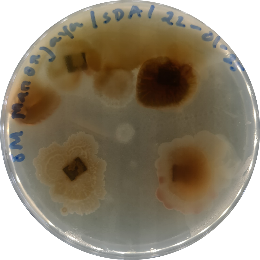 |
|  |  |  |  | **Reinoculated culture – recovered isolates**  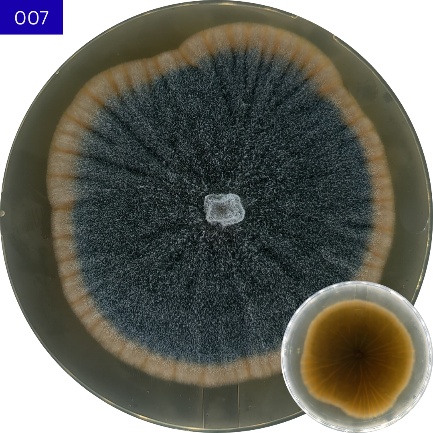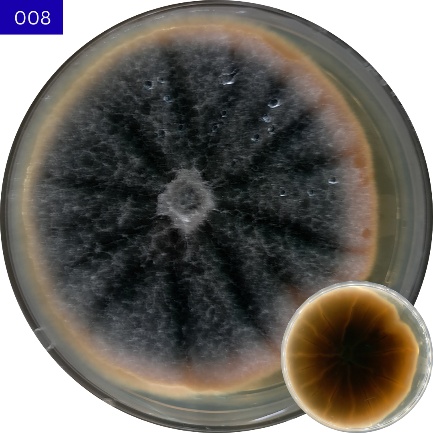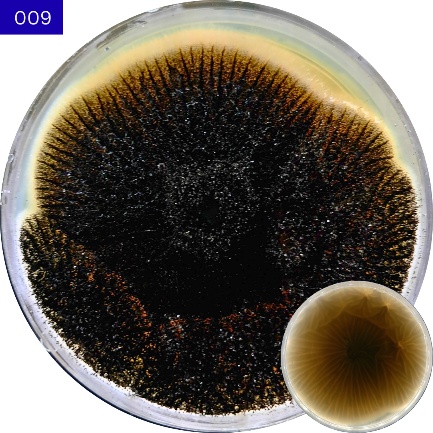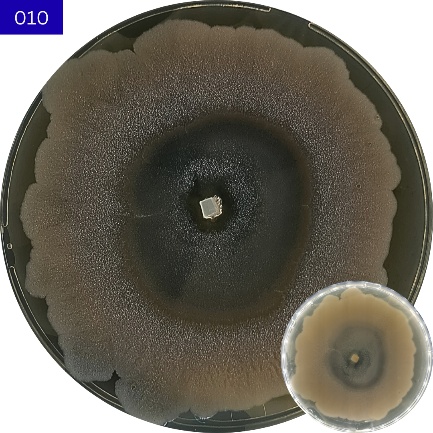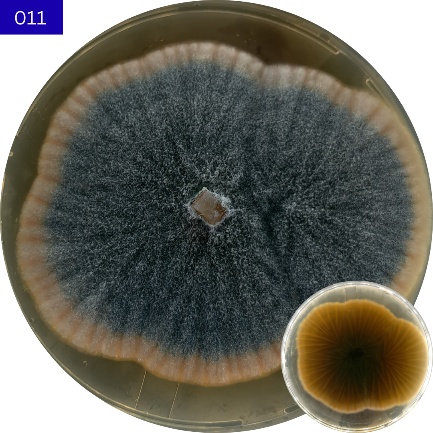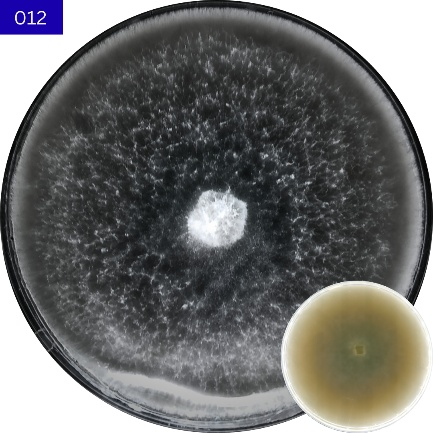 | | | |

| **No** | **Code** | **Location** | **Leaf Picture** | **Saboraud Dextrose Agar (SDA) + chloramphenicol (0.4 µg/mL) Duplicated Innoculation** | | | |
| --- | --- | --- | --- | --- | --- | --- | --- |
|  |  |  |  | **Upper** | **Reverse** | **Upper** | **Reverse** |
| 3 | SM1003 | Padakembang | 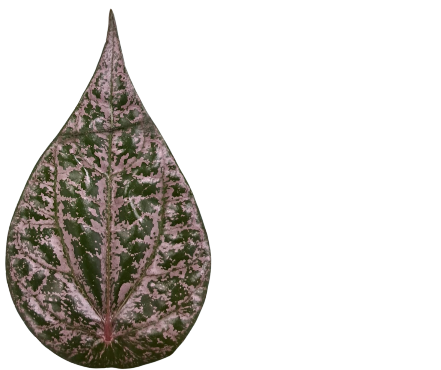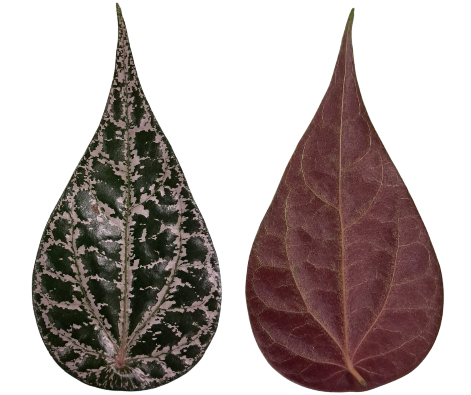 | 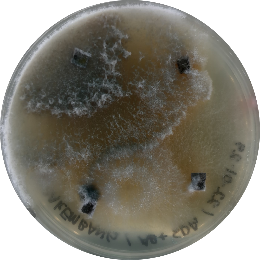 | 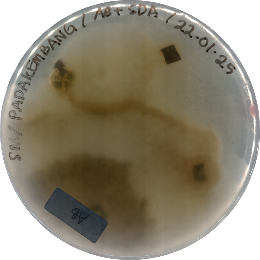 | 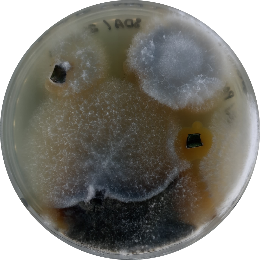 | 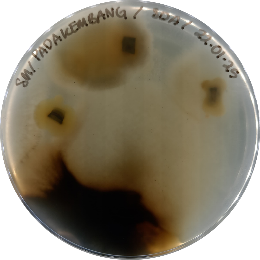 |
|  |  |  |  | **Reinoculated culture – recovered isolates**  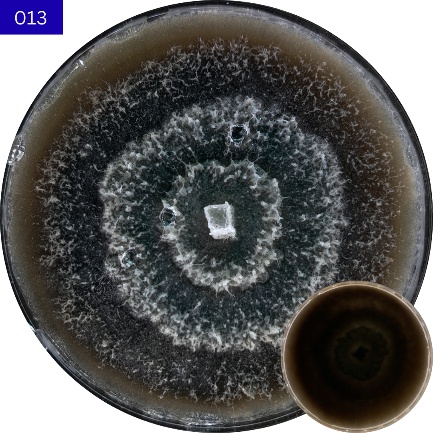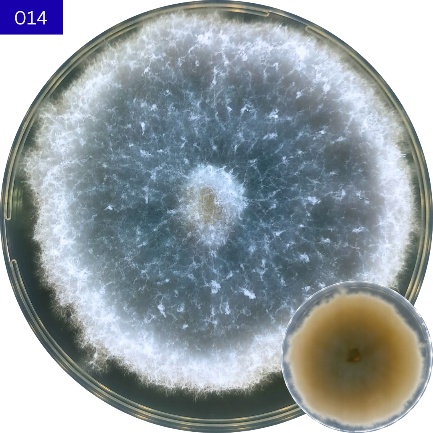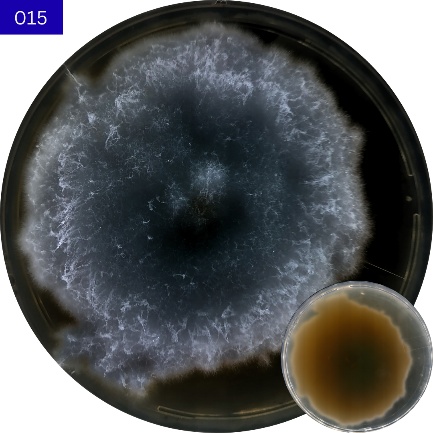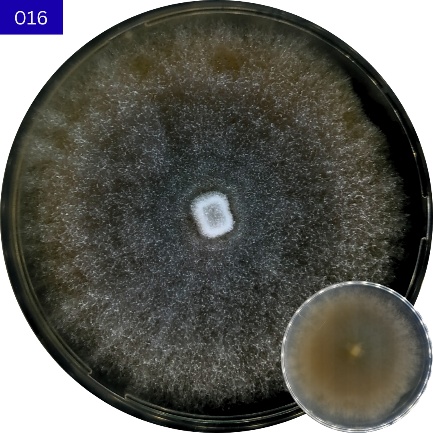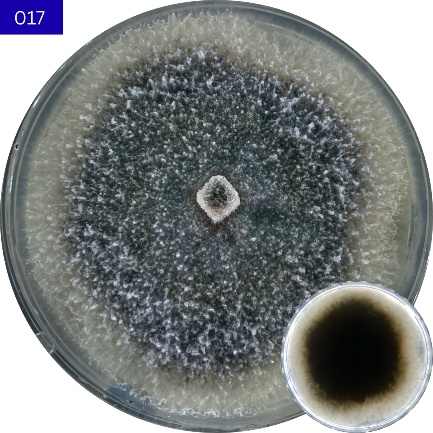 | | | |

| **No** | **Code** | **Location** | **Leaf Picture** | **Saboraud Dextrose Agar (SDA) + chloramphenicol (0.4 µg/mL) Duplicated Innoculation** | | | |
| --- | --- | --- | --- | --- | --- | --- | --- |
|  |  |  |  | **Upper** | **Reverse** | **Upper** | **Reverse** |
| 4 | SM1004 | Sukaratu | 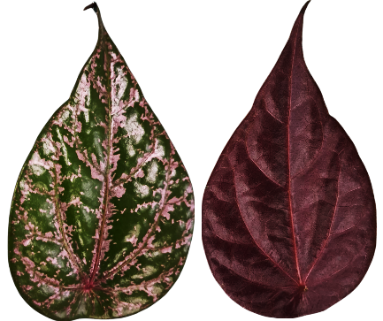 | 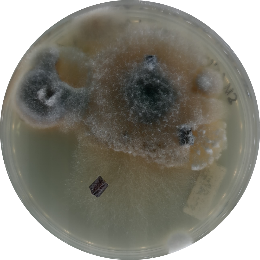 | 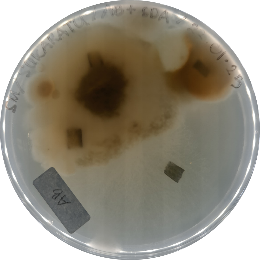 | 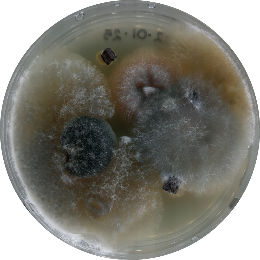 | 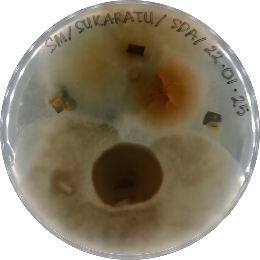 |
|  |  |  |  | **Reinoculated culture – recovered isolates**  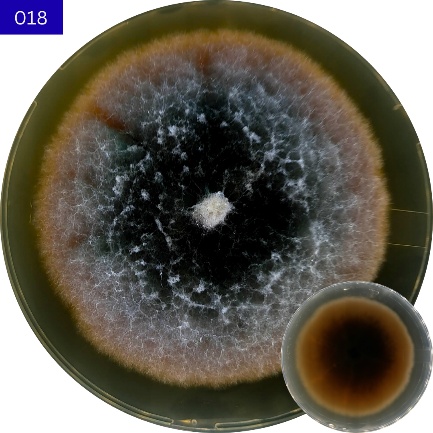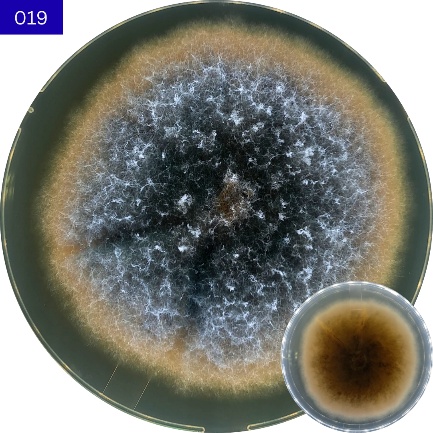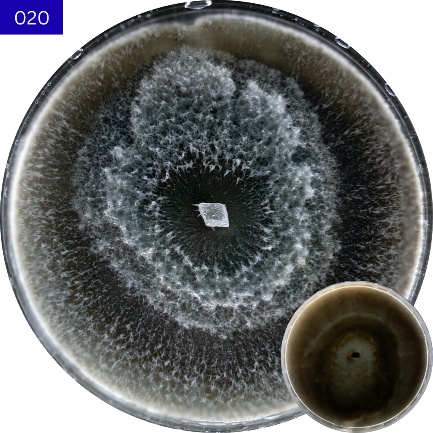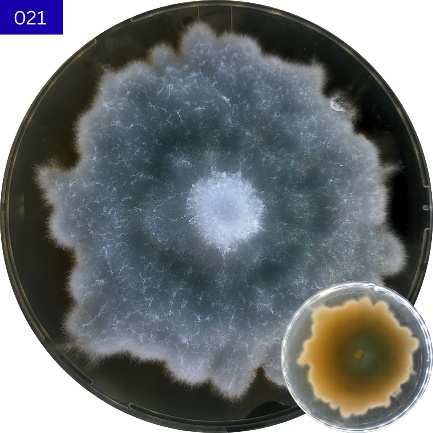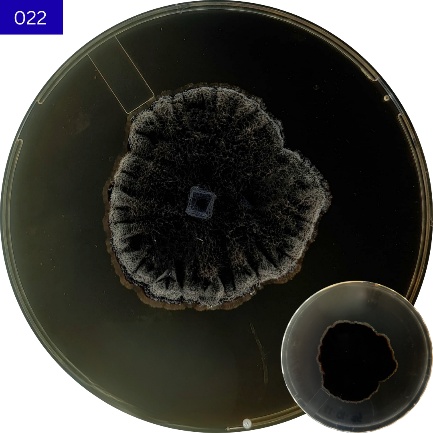 | | | |

| **No** | **Code** | **Location** | **Leaf Picture** | **Saboraud Dextrose Agar (SDA) + chloramphenicol (0.4 µg/mL) Duplicated Innoculation** | | | |
| --- | --- | --- | --- | --- | --- | --- | --- |
|  |  |  |  | **Upper** | **Reverse** | **Upper** | **Reverse** |
| 5 | SM1005 | Singaparna | 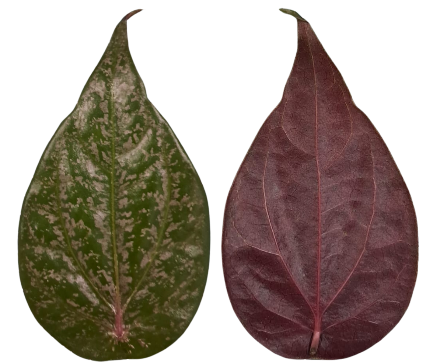 | 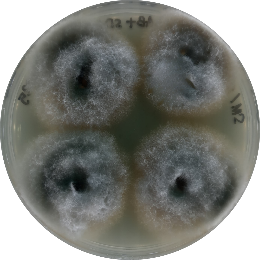 | 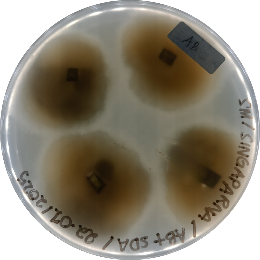 | 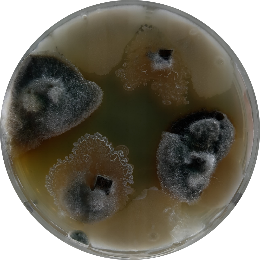 | 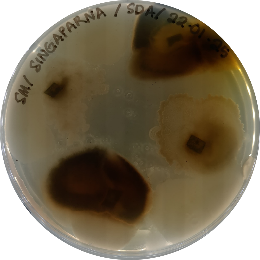 |
|  |  |  |  | **Reinoculated culture – recovered isolates**  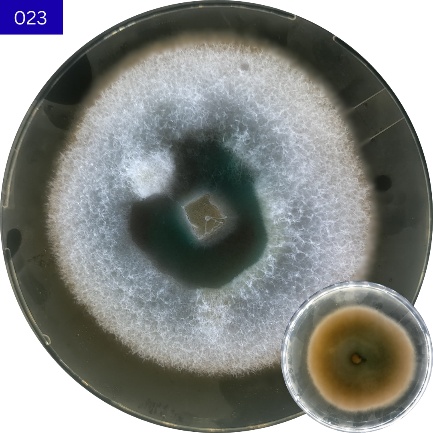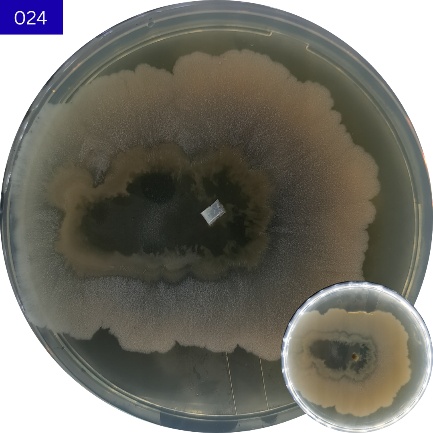  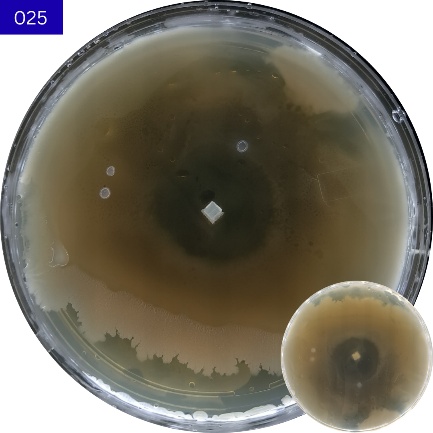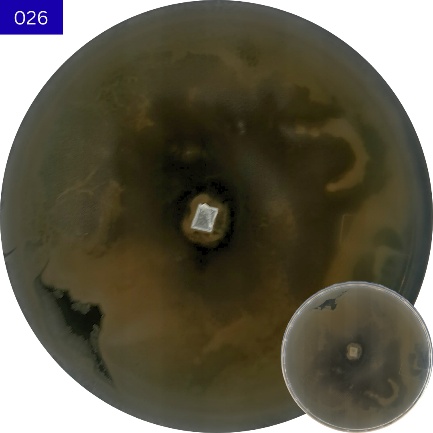 | | | |

| **No** | **Code** | **Location** | **Leaf Picture** | **Saboraud Dextrose Agar (SDA) + chloramphenicol (0.4 µg/mL) Duplicated Innoculation** | | | |
| --- | --- | --- | --- | --- | --- | --- | --- |
|  |  |  |  | **Upper** | **Reverse** | **Upper** | **Reverse** |
| 6 | SM1006 | Lanud | 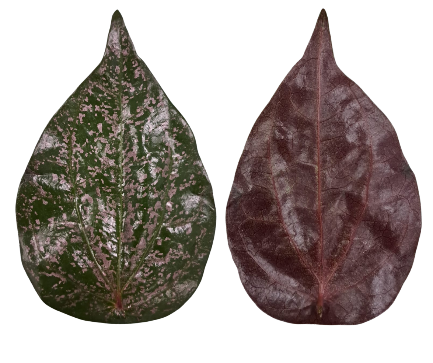 | 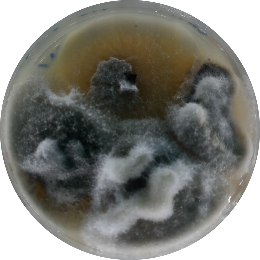 | 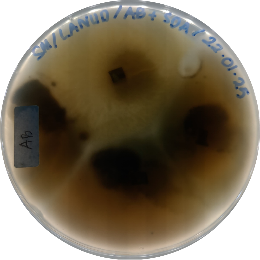 | 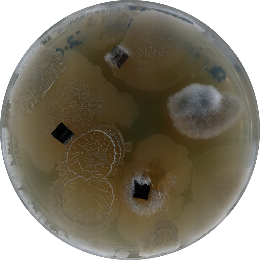 | 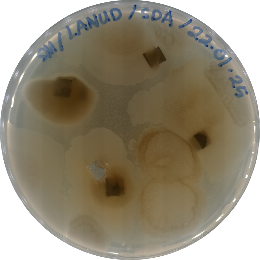 |
|  |  |  |  | **Reinoculated culture – recovered isolates**  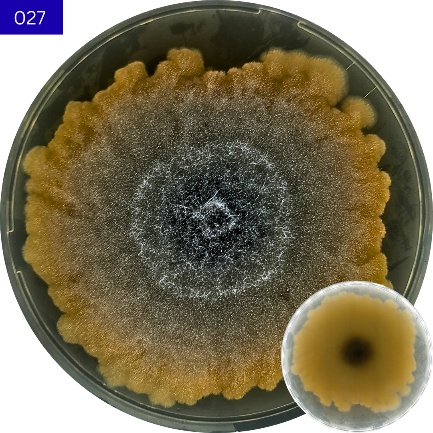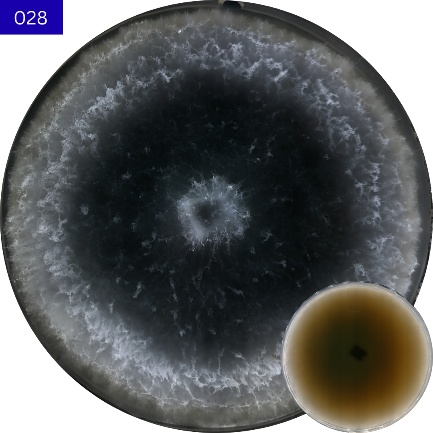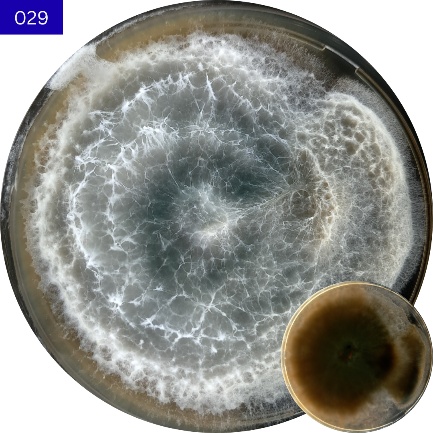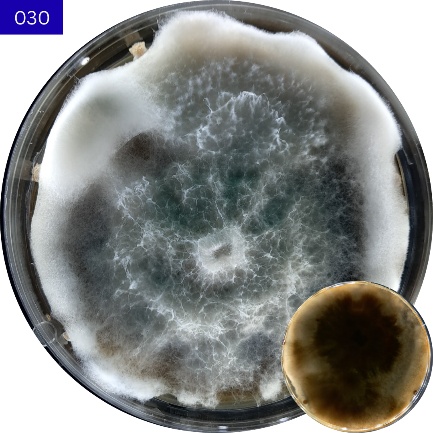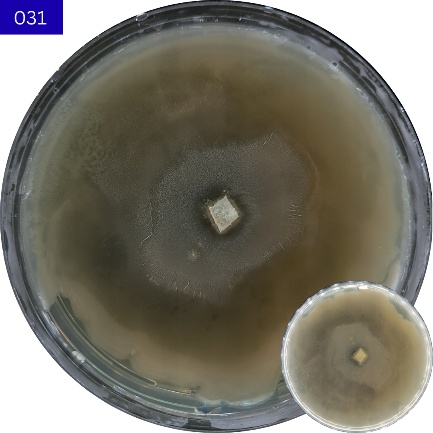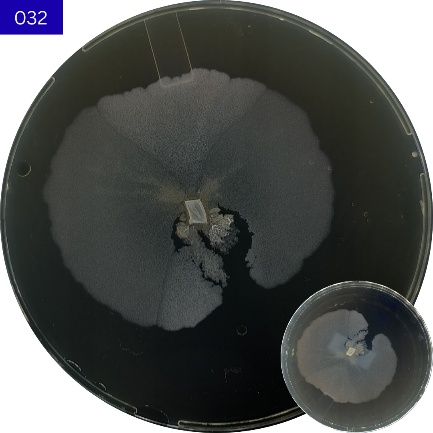 | | | |

| **No** | **Code** | **Location** | **Leaf Picture** | **Saboraud Dextrose Agar (SDA) + chloramphenicol (0.4 µg/mL) Duplicated Innoculation** | | | |
| --- | --- | --- | --- | --- | --- | --- | --- |
|  |  |  |  | **Upper** | **Reverse** | **Upper** | **Reverse** |
| 7 | SM2007 | Singajaya | 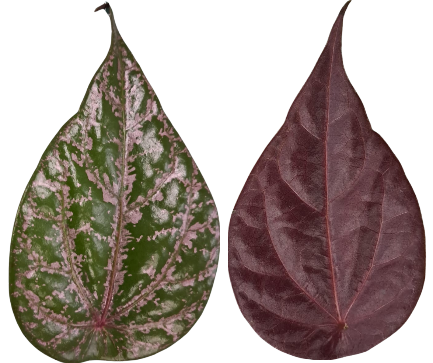 | 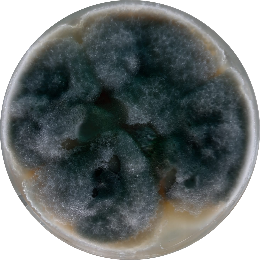 | 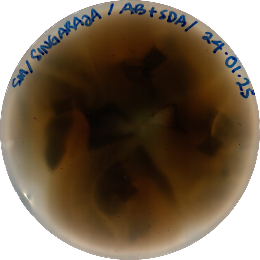 | 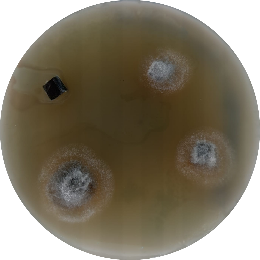 | 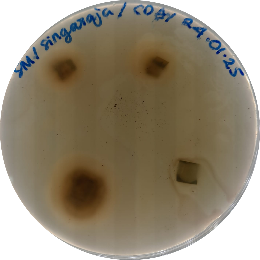 |
|  |  |  |  | **Reinoculated culture – recovered isolates**  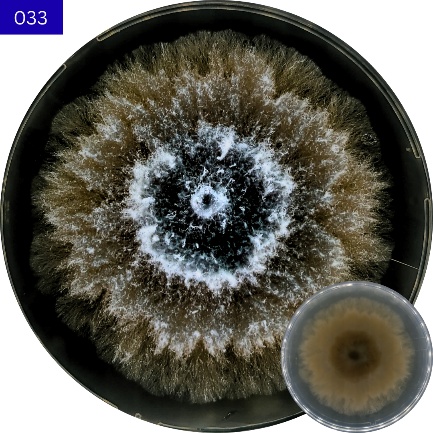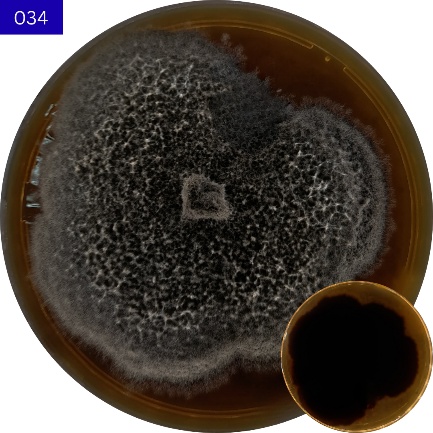  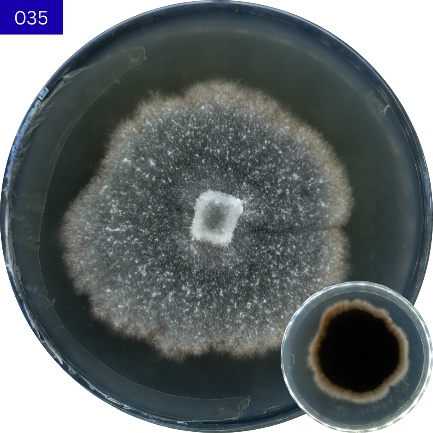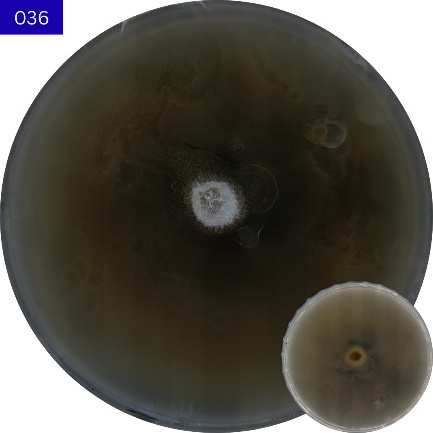 | | | |

| **No** | **Code** | **Location** | **Leaf Picture** | **Saboraud Dextrose Agar (SDA) + chloramphenicol (0.4 µg/mL) Duplicated Innoculation** | | | |
| --- | --- | --- | --- | --- | --- | --- | --- |
|  |  |  |  | **Upper** | **Reverse** | **Upper** | **Reverse** |
| 8 | SM2008 | Kubangsari | 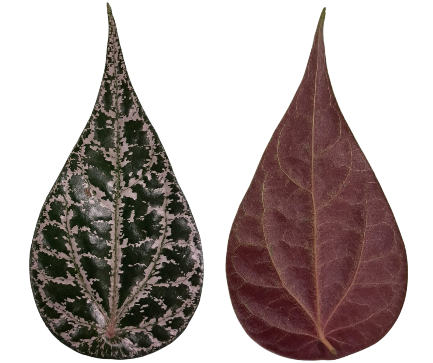 | 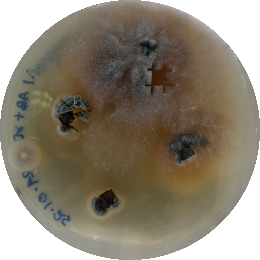 | 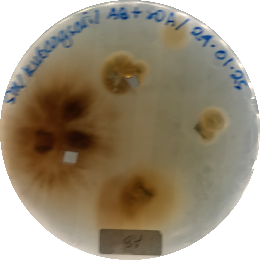 | 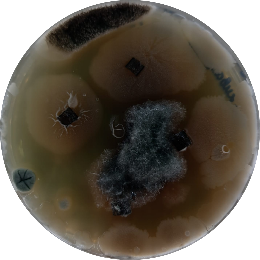 | 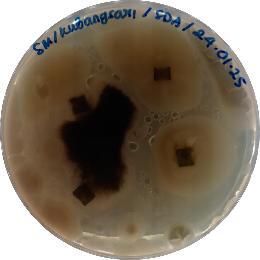 |
|  |  |  |  | **Reinoculated culture – recovered isolates**  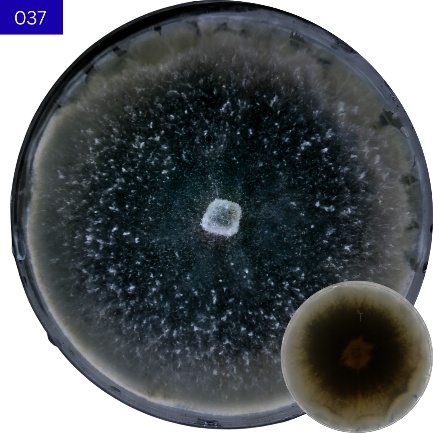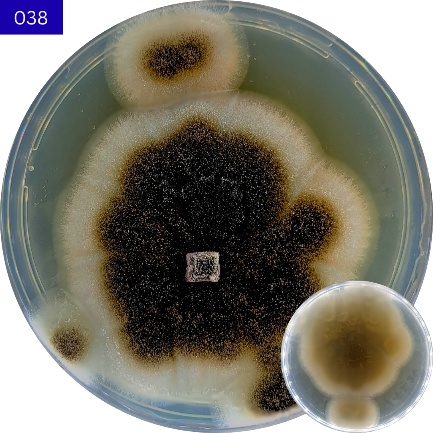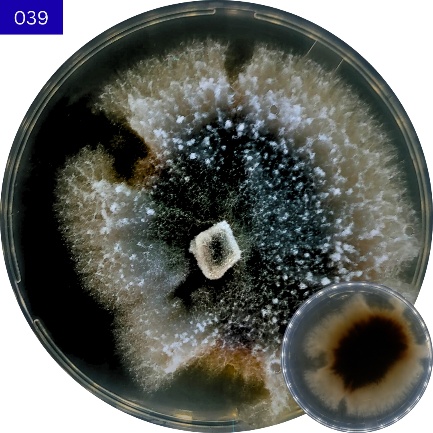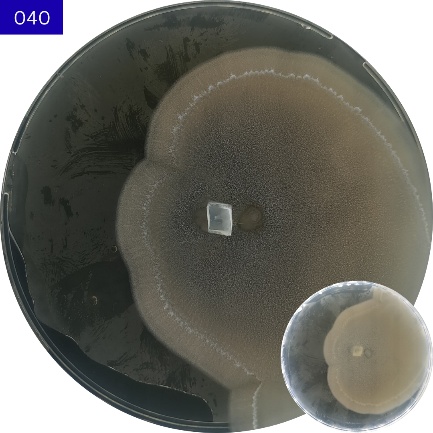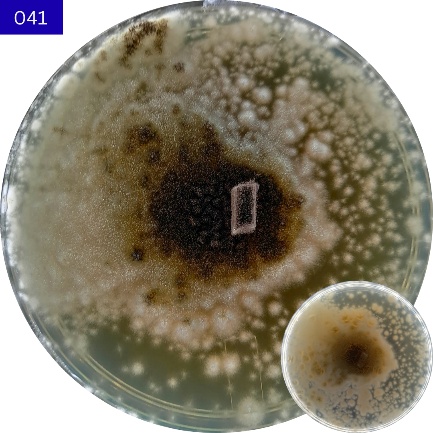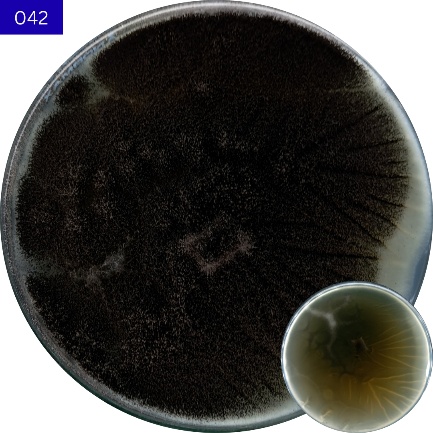 | | | |

| **No** | **Code** | **Location** | **Leaf Picture** | **Saboraud Dextrose Agar (SDA) + chloramphenicol (0.4 µg/mL) Duplicated Innoculation** | | | |
| --- | --- | --- | --- | --- | --- | --- | --- |
|  |  |  |  | **Upper** | **Reverse** | **Upper** | **Reverse** |
| 9 | SM2009 | Setiaratu | 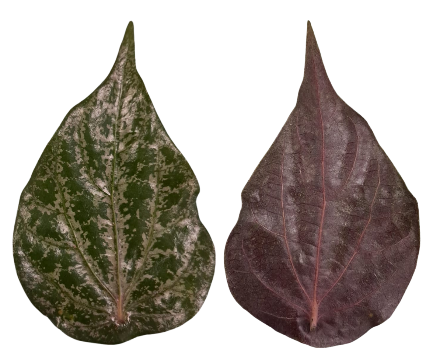 | 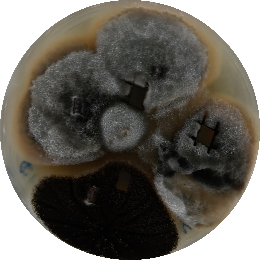 | 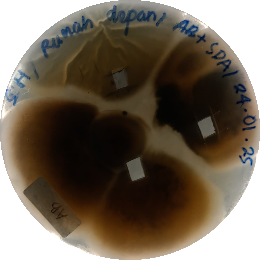 | 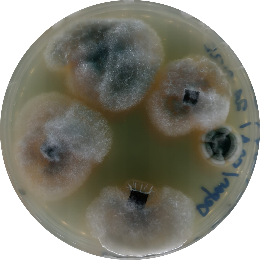 | 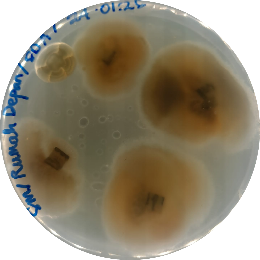 |
|  |  |  |  | **Reinoculated culture – recovered isolates**  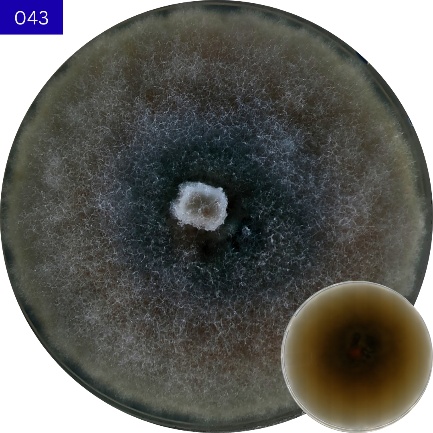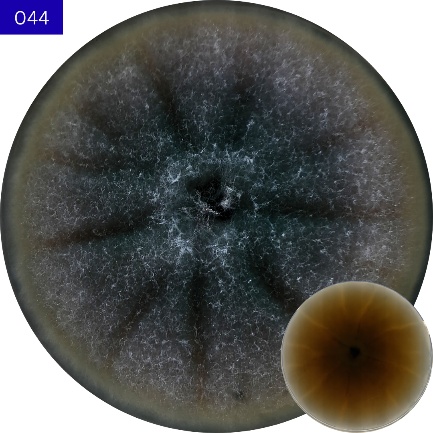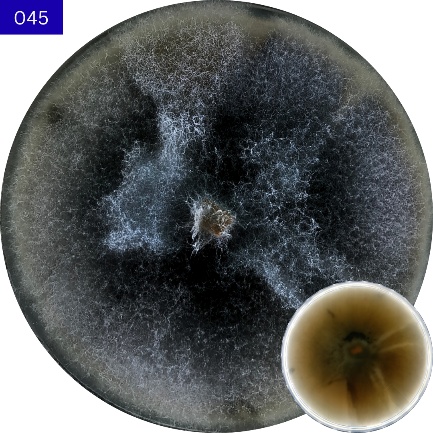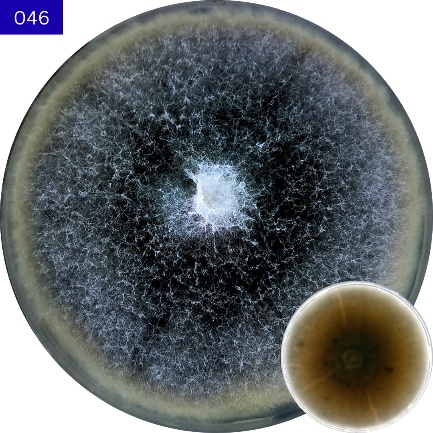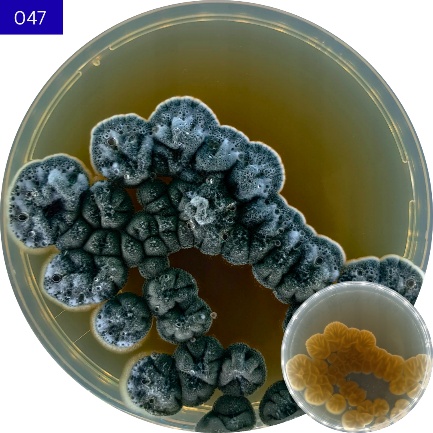 | | | |

| **No** | **Code** | **Location** | **Leaf Picture** | **Saboraud Dextrose Agar (SDA) + chloramphenicol (0.4 µg/mL) Duplicated Innoculation** | | | |
| --- | --- | --- | --- | --- | --- | --- | --- |
|  |  |  |  | **Upper** | **Reverse** | **Upper** | **Reverse** |
| 10 | SM2010 | Cibeureum | 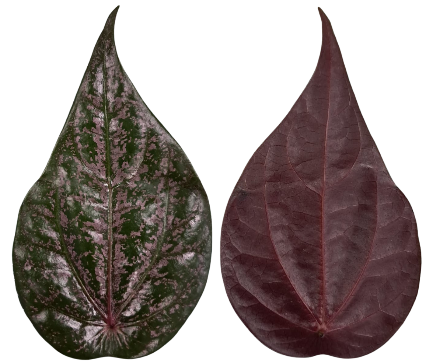 | 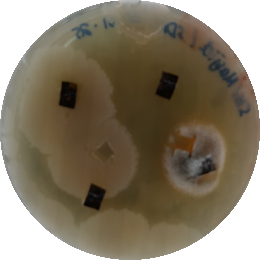 | 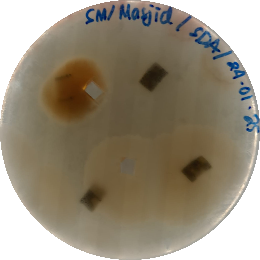 | 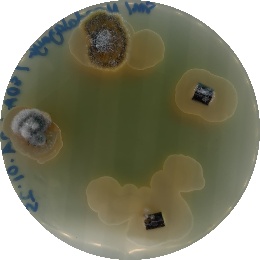 | 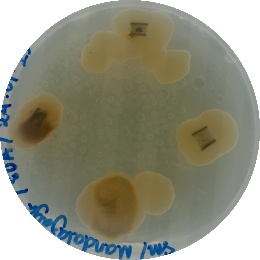 |
|  |  |  |  | **Reinoculated culture – recovered isolates**  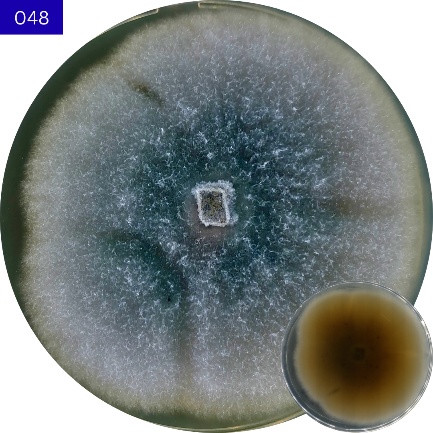 | | | |

| **No** | **Code** | **Location** | **Leaf Picture** | **Saboraud Dextrose Agar (SDA) + chloramphenicol (0.4 µg/mL) Duplicated Innoculation** | | | |
| --- | --- | --- | --- | --- | --- | --- | --- |
|  |  |  |  | **Upper** | **Reverse** | **Upper** | **Reverse** |
| 11 | SM2011 | Cisalak |  |  |  |  |  |
|  |  |  |  | **Reinoculated culture – recovered isolates** | | | |

| **No** | **Code** | **Location** | **Leaf Picture** | **Saboraud Dextrose Agar (SDA) + chloramphenicol (0.4 µg/mL) Duplicated Innoculation** | | | |
| --- | --- | --- | --- | --- | --- | --- | --- |
|  |  |  |  | **Upper** | **Reverse** | **Upper** | **Reverse** |
| 12 | SM2012 | Mandalajaya |  |  |  |  |  |
|  |  |  |  | **Reinoculated culture – recovered isolates** | | | |

| **No** | **Code** | **Location** | **Leaf Picture** | **Saboraud Dextrose Agar (SDA) + chloramphenicol (0.4 µg/mL) Duplicated Innoculation** | | | |
| --- | --- | --- | --- | --- | --- | --- | --- |
|  |  |  |  | **Upper** | **Reverse** | **Upper** | **Reverse** |
| 13 | SM2013 | Tamansari |  |  |  |  |  |
|  |  |  |  | **Reinoculated culture – recovered isolates** | | | |

**Table S3.** List of selected endophytic fungal isolates from *Piper crocatum* and their GenBank accession numbers based on ITS sequences

| **No** | **Sampling source** | **Isolate code** | **Tentative species identification** | **Marker** | **GenBank accession number** | **BP length** | **Submission ID** |
| --- | --- | --- | --- | --- | --- | --- | --- |
| 1 | *Piper crocatum* leaf tissue from Mandalajaya, Tasikmalaya, West Java | SM_059_124A | *Colletotrichum truncatum* | ITS (rRNA region) | PX415253 | 555 | SUB15615992 |
|  | > SM_059_124A  CCTGCGGAGGGATCATTACTGAGTTACCGCTCATCAACCCTTTGTGAACATACCTTAACTGTTGCTTCGGCGGGTAGGCGTCCCCTGAAAAGGACGTCTCCCGGCCCTCTCCCGTCCGCGGGTGGGGCGCCCGCCGGAGGATAACCAAACTCTGATTTAACGACGTTTCTTCTGAGTGACACAAGCAAATAATCAAAACTTTTAACAACGGATCTCTTGGTTCTGGCATCGATGAAGAACGCAGCGAAATGCGATAAGTAATGTGAATTGCAGAATTCAGTGAATCATCGAATCTTTGAACGCACATTGCGCCCGCCAGCATTCTGGCGGGCATGCCTGTTCGAGCGTCATTTCAACCCTCAAGCTCTGCTTGGTGTTGGGGCTCTACGGTTGACGTAGGCCCTTAAAGGTAGTGGCGGACCCTCTCGGAGCCTCCTTTGCGTAGTAACATTTCGTCTCGCATTGGGATTCGGAGGGACTCTAGCCGTAAAACCCCCAATTTTACTAAGGTTGACCTCGGATCAGGTAGGAATACCCGCTGAACTTAAGCATATC | | | | | | |
| 2 | *Piper crocatum* leaf tissue from Manonjaya, Tasikmalaya, West Java | SM_007_012 | *Aspergillus clavatonicus* | ITS (rRNA region) | PX415421 | 580 | SUB15665397 |
|  | > SM_007_012  ACCTGCGGAAGGATCATTACCGAGTGCGGGCCCTCTGGGTCCAACCTCCCACCCGTGTCTATTGTACCTTGTTGCTTCGGCGGGCCCGCCGTCTTCGGACGGCCGCCGGGGAGGCCTCCGCGCCCCCGGGCCCGCGCCCGCCGAAGACCACAACATGAACTCTGTTCTGAAGTTTTGCAGTCTGAGTTGATTATCATAATCAGTTAAAACTTTCAACAACGGATCTCTTGGTTCCGGCATCGATGAAGAACGCAGCGAAATGCGATAACTAATGTGAATTGCAGAATTCAGTGAATCATCGAGTCTTTGAACGCACATTGCGCCCCCTGGTATTCCGGGGGGCATGCCTGTCCGAGCGTCATTGCTGCCCTCAAGCACGGCTTGTGTGTTGGGCCCCCGTCCCCGCCTCACCGCGGGGACGGGCCCGAAAGGCAGCGGCGGCACCGCGTCCGGTCCTCGAGCGTATGGGGCTTTGTCACCCGCTCTTGTAGGCCCGGCCGGCGCCTGTCGACACCAACCCCAATTTTTCTAAGGTTGACCTCGGATCAGGTAGGGATACCCGCTGAACTTAAGCATATCA | | | | | | |
| 3 | *Piper crocatum* leaf tissue from Mandalajaya, Tasikmalaya, West Java | SM_062_123 | *Colletotrichum cliviae* | ITS (rRNA region) | PX415427 | 554 | SUB15665469 |
|  | > SM_062_123  CCTGCGGAGGGATCATTATCGAGTTACCGCTCCTTATAACCCTTTGTGAACATACCCCAAACGTTGCCTCGGCGGGCAGCCGGAGCCCAGCTCCGTCGCCCGGAGCCGCCGTCTCGGCGCGCCCCACCCGCCGGCGGACCACCAAACTCTATTTAAACGACGTCTCTTCTGAGTGGCACAAGCAAATAATCAAAACTTTTAACAACGGATCTCTTGGTTCTGGCATCGATGAAGAACGCAGCGAAATGCGATAAGTAATGTGAATTGCAGAATTCAGTGAATCATCGAATCTTTGAACGCACATTGCGCCCGCCAGCATTCTGGCGGGCATGCCTGTTCGAGCGTCATTTCAACCCTCAAGCACCGCTTGGCGTTGGGGCCCTACGGCTTCCGTAGGCCCCGAAATACAGTGGCGGACCCTCCCGGAGCCTCCTTTGCGTAGTAACATACCACCTCGCACTGGGATCCGGAGGGACTCCTGCCGTAAAACCCCCCAATTTTCCAAAGGTTGACCTCGGATCAGGTAGGAATACCCGCTGAACTTAAGCATATCA | | | | | | |
| 4 | *Piper crocatum* leaf tissue from Cisalak, Tasikmalaya, West Java | SM_052_118 | *Torula canangae* | ITS (rRNA region) | PX415440 | 551 | SUB15665794 |
|  | > SM_052_118  CTTCCGTAGGGGAACCTGCGGAAGGATCATTAGAGAAATGAGGCGAAGGGCGGTCCAACCGCCGCCCGGAGCCGACACTCTCCACCCTGCCTTTTGAATACCTCACCTCCTCCCCGGCCTCCGCGCCGGGCTGGCTTACTTGAAAAAACCTTGCAGTTATAGTCCATTCAGTCATTACAAACAAAATTACAACTTTCAACAATGGATCTCTTGGCTCTGGCATCGATGAAGAACGCAGCGAAATGCGATAAGTAGTGTGAATTGCAGAATTCAGCGAATCATCGAATCTTTGAACGCACATTGCGCCCTTCGGTTATTCCTTAGGGCATGCCTGTTCGAGCGTCATTTCAACCTTCAAGCCTGGCTTGGTGTTGGGCGCTGTCCCGCCCCCGCGCGCGGACTCGCCTCGAATGAATTGGCAGTCGCACCCTCCGAGCCGCGAGCGCAGCACAAGTCGCGCCCGCCGGTCCCGCTGGGACGGACGCTCCACAAGACCCCCCACAGTCTTGACCTCGGATCAGGTAGGGATACCCGCTGAACTTAAGCATATC | | | | | | |

K - K+ Ladder 007

K - K+ Ladder 052

B

A

K - K+ Ladder 062

C

D
